# Activity of the SWI/SNF complex is indispensable for syncytiotrophoblast formation

**DOI:** 10.1242/dev.204770

**Published:** 2025-10-31

**Authors:** Henrieta Papuchova, Andreas Lackner, Terezia Vcelkova, Petra Tolp, Sandra Haider, Vasileios Gerakopoulos, Paulina A. Latos

**Affiliations:** ^1^ Division of Cell and Developmental Biology, Center for Anatomy and Cell Biology, Medical University of Vienna, A-1090 Vienna, Austria; ^2^ Placental Development Group, Reproductive Biology Unit, Department of Obstetrics and Gynaecology, Medical University of Vienna, A-1090 Vienna, Austria

**Keywords:** Trophoblast stem cells, Placenta, SWI/SNF

## Abstract

Developmental transitions are characterized by coordinated changes in lineage-specific gene expression programs and chromatin states. Yet how these shifts in cell fate occur during placental development remains largely unknown. Here, we have used human trophoblast stem cells (hTSCs), genetic depletion and small-molecule inhibition of the SWI/SNF remodelling complex activity to address its role during syncytiotrophoblast (ST) differentiation. We found that SWI/SNF inhibition has a massive impact on gene expression, chromatin accessibility and histone modifications, particularly H3K27ac, resulting in ST differentiation failure. We also observed cell cycle defects, indicating that SWI/SNF is required for hTSCs to exit the cell cycle, which is a prerequisite for ST commitment. In addition, based on motif analysis of SWI/SNF target regions, we genetically tested several early ST candidate transcription factors. While GCM1, CEBPB and TBX3 are vital for ST differentiation, only GCM1 is sufficient to induce ST fate. Together, our results demonstrate that SWI/SNF activity is essential for lineage specification during placental development.

## Teaser

**Summary:** SWI/SNF drives global chromatin and gene expression changes during human trophoblast stem cell differentiation and is essential for syncytiotrophoblast formation.

## INTRODUCTION

The human placenta plays an essential role in supporting foetal development, ensuring the exchange of gases, nutrients and metabolites, and hormone production and immune regulation. Integral to its functionality are specialized trophoblast cell types emerging during embryonic development from a progenitor population called the cytotrophoblast (CT). The CT gives rise to invasive extravillous trophoblast (EVT) with immune-modulatory properties and to the multinuclear syncytiotrophoblast (ST). The ST has a prominent endocrine function, producing hormones for pregnancy maintenance, and serves as a primary interface between the foetus and the mother (Knöfler et al., 2019; Turco and Moffett, 2019). ST differentiation is driven by the coordinated action of signalling pathways (e.g. PKA/cAMP, MAPK and PKC), transcription factors (e.g. GCM1, CREB, OVOL1 and TFAP2A) (Papuchova and Latos, 2022) and cooperating histone modifiers (e.g. HDAC1/2, p300/CPB and MLL1) (Jaju Bhattad et al., 2020; van Voorden et al., 2023; Wu et al., 2024), resulting in cell cycle exit and the shutdown of the CT program, and turning on the ST gene expression signature (Jaremek et al., 2021; Lu et al., 2017; Renaud and Jeyarajah, 2022). However, how transcription factors cooperate with chromatin remodelling during ST differentiation remains unexplored.

ATP-dependent control of chromatin accessibility through nucleosome repositioning is a crucial step in the transcriptional regulation of cell identity. By shifting and ejecting nucleosomes, the chromatin remodelling complexes facilitate the recruitment of transcriptional factors, regulatory proteins and the RNAPII transcriptional machinery (Brahma and Henikoff, 2024). Chromatin remodelling-controlled local and global genome reorganization profoundly influences gene expression and differentiation by regulating enhancer activity, transcriptional initiation, elongation and termination, as well as pre-mRNA splicing (Alver et al., 2017; Basurto-Cayuela et al., 2024). Chromatin accessibility in mammals is regulated by the four major families of nucleosome remodelling complexes: SWI/SNF (switch/sucrose non-fermentable), ISWI (imitation switch), CDH (chromodomain helicase DNA binding) and INO80 (named after inositol requiring 80 kDa protein in *S. cerevisiae*) (Bowman, 2025; Centore et al., 2020; Clapier et al., 2017; Goodwin and Picketts, 2018; Marfella and Imbalzano, 2007). They consist of a catalytic ATPase and accessory subunits, and are frequently mutated in cancer (Eustermann et al., 2024; Mittal and Roberts, 2020). The SWI/SNF family encompasses three distinct complexes: canonical (cBAF), non-canonical (ncBAF) and polybromo-associated BAF (PBAF) that contain one of the two ATPases BRM (SMARCA2) or BRG1 (SMARCA4), along a variety of accessory subunits (Centore et al., 2020; Kadoch and Crabtree, 2015). SWI/SNF has been identified as a key regulator of gene expression and cell fate decisions (Ho et al., 2009). It has been shown to control self-renewal and differentiation of human and murine embryonic stem cells (ESCs), including into the cardiac (Hota et al., 2022) and neural lineages (Ye et al., 2021; Zhang et al., 2014). Despite the crucial roles of SWI/SNF in cell fate decisions, its involvement in human trophoblast differentiation, particularly ST formation, which is characterized by rapid cell fate changes, remains to be explored.

Here, we have investigated the function of the SWI/SNF complex during CT to ST differentiation using human trophoblast stem cells (hTSCs) as an *in vitro* model (Okae et al., 2018). We demonstrate that SWI/SNF inhibition dysregulates gene expression, the cell cycle, chromatin accessibility and histone modifications, leading to a ST differentiation defect.

## RESULTS

### BRM/BRG1 inhibition leads to ST differentiation defect

**Fig. 1.**
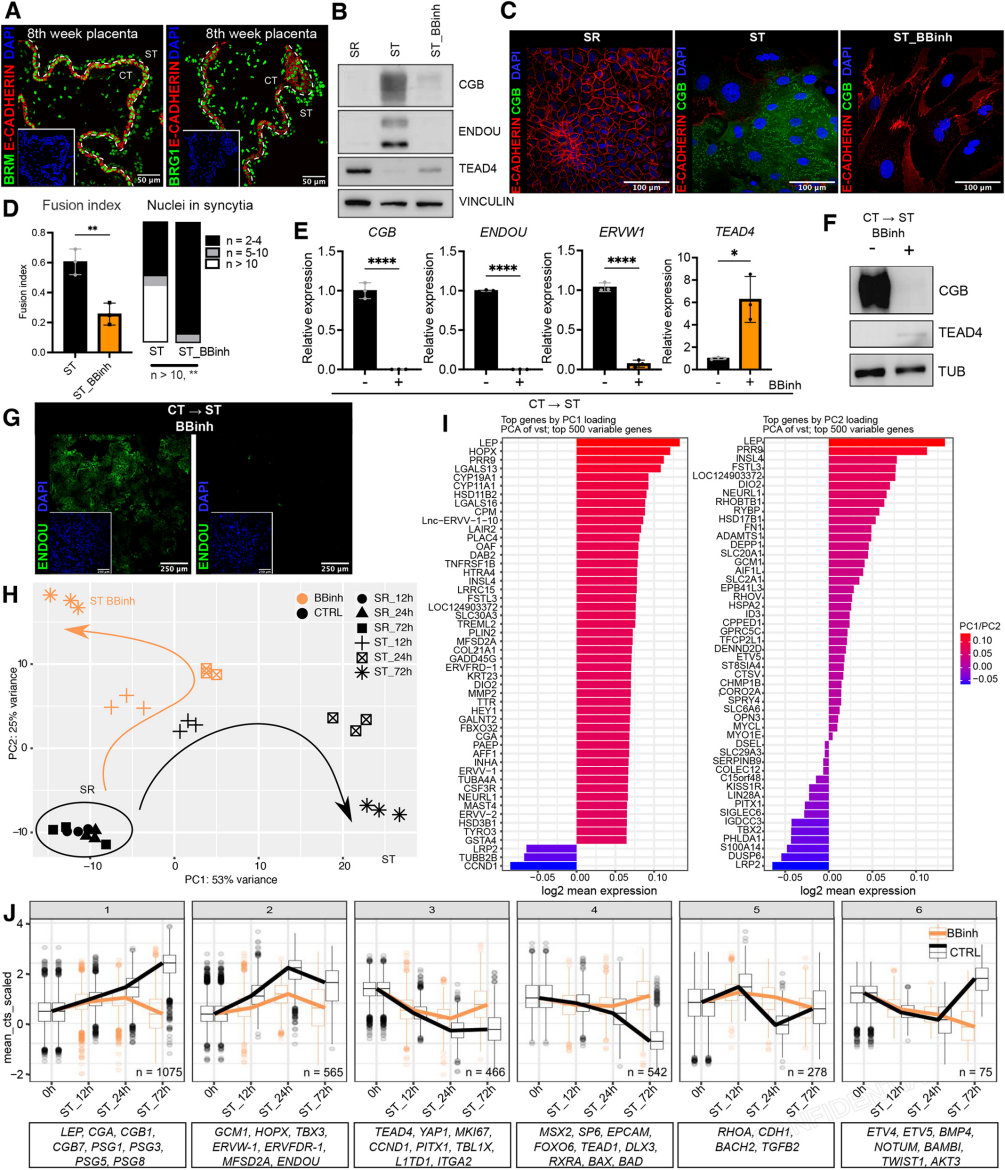
**BRM/BRG1 inhibition leads to syncytiotrophoblast differentiation defects.** (A) Immunofluorescence staining of BRM or BRG1 (green), E-cadherin (red) and DAPI (blue) in placental sections (8th week). (B) Western blot of self-renewing (SR), syncytiotrophoblast (ST) and BBinh-treated ST cells (72 h) showing CGB, ENDOU and TEAD4. Vinculin serves as a loading control. (C) Immunofluorescence staining of E-cadherin (red), CGB (green) and DAPI (blue) in SR, ST and BBinh-treated ST cells. The distinct morphology of ST_BBinh compared to SR cells was likely caused by different culture conditions (ST versus SR). See also Fig. 6C. (D) Quantification of fusion index [fusion index=(number of nuclei in syncytia−number of syncytia)/total number of nuclei]. Syncytium was defined by two or more nuclei. **P<0.01, unpaired *t*-test (data are mean±s.e.m.); *n*=3, 3 areas, 2 independent observers, 72 h. (E) RT-qPCR analysis of freshly isolated villous cytotrophoblast (CT) (6th-7th week) spontaneously differentiating to ST treated with (+) and without (−) BBinh (72 h). *P<0.05, ****P<0.0001, unpaired *t*-test (data are mean±s.e.m.), *n*=3. (F) Western blot of freshly isolated villous CT (6th-7th week) spontaneously differentiating into ST treated with (+) or without (−) BBinh (72 h). Tubulin serves as a loading control. (G) Immunofluorescence staining of ENDOU (green) and DAPI (blue) in freshly isolated villous CT spontaneously differentiating into ST treated with (+) or without (−) BBinh (72 h). (H) PCA plot based on global gene expression (QuantSeq) in SR, ST and BBinh-treated ST after 12, 24 and 72 h of differentiation/treatment (*n*=3). (I) Top 50 genes sorted by PC1 loading (left) or PC2 loading (right) (related to H). (J) Line plots of aggregated gene expression (scaled variance stabilizing transformation counts) of six clusters based on hierarchical clustering of the 3000 most variable genes during ST differentiation of untreated hTSCs.

Previously, we have shown that cBAF, ncBAF and PBAF complexes operate in hTSCs, and we demonstrated the interaction of cBAF with the MSX2 transcription factor (TF). Since the depletion of MSX2 led to spontaneous ST differentiation and increased occupancy of cBAF and H3K27ac, we speculated that cBAF may be involved in the regulation of ST differentiation (Hornbachner et al., 2021). Here, we test this hypothesis during hTSC to ST differentiation. While self-renewing hTSCs represent the CT population of the human placenta, upon induction with forskolin, they rapidly differentiate to the ST (Okae et al., 2018). To address the function of SWI/SNF, we utilized the highly specific and extensively characterized dual allosteric inhibitor BRM014 (hereafter referred to as BBinh), which inhibits the ATPase activity of both BRM and BRG1 (Basurto-Cayuela et al., 2024; Iurlaro et al., 2021; Martin et al., 2023; Papillon et al., 2018; Schick et al., 2021). Of note, both BRM and BRG1 are expressed in hTSCs, and in the CT and ST (Fig. 1A, Fig. S1A) (Hornbachner et al., 2021). The BBinh has been used previously, providing new insights into the workings of SWI/SNF in different cellular contexts, including murine embryonic stem cells (mESCs) (Iurlaro et al., 2021; Papillon et al., 2018; Schick et al., 2021). Differentiation of hTSCs into the ST was induced by 2 µM forskolin for 72 h, either in the presence or absence of 10 µM BBinh (Fig. S1B). While the control cells downregulated the self-renewal (SR) regulators (TEAD4) and concomitantly upregulated ST markers (CGB and ENDOU), the BBinh-treated cells failed to differentiate to ST (Fig. 1B, Fig. S10). Similarly, while the control cells fused, forming multinucleated syncytia, the BBinh-treated cells were unable to downregulate E-cadherin and fuse (Fig. 1C,D). To independently validate the effects of BBinh, we asked whether depletion of BRM and BRG1 would phenocopy inhibition of their catalytic activities. Using the lentiviral-mediated shRNA knockdown (KD) approach, we generated BRM_KD, BRG1_KD, the double (d) BRM/BRG1_dKD and the control (CTRL_KD) TSC lines, and induced their differentiation into ST. Gene expression analysis revealed that differentiation of the BRM_KD and BRG1_KD lines was indistinguishable from the control (CTRL_KD), likely due to the redundancy of the BRM and BRG1 proteins (Fig. S1C,D, Fig. S10). In stark contrast, the BRM/BRG1_dKD showed a strong ST differentiation defect that was reminiscent of the BBinh_ST phenotype. Similar to BBinh_ST, dKD_ST failed to upregulate key ST markers (CGB and ENDOU) and was unable to downregulate the SR marker TEAD4 at both the RNA and protein levels (Fig. S1C,D). Global gene expression changes in dKD_ST, as examined by 3′mRNA-seq, matched those of BBinh_ST, as these samples clustered closely together in the PCA plot and showed a significant correlation in their transcriptional profiles (Fig. S1E,F). Finally, Gene Ontology (GO) analysis revealed female pregnancy, mitotic nuclear division and chromosome segregation among the terms enriched in dKD_ST downregulated genes, confirming the ST differentiation defects (Fig. S1G). To further validate our findings, we employed alternative trophoblast models, including primary trophoblast cells, trophoblast organoids (TBorg) and a 3D ST-differentiation system. Villous CT, freshly isolated from 1st-trimester human placentas, spontaneously fuses and differentiates into ST when cultured in 10% FBS/DMEM. Following 72 h of treatment with BBinh, the CT failed to upregulate the ST markers CGB and ENDOU, to downregulate the SR marker TEAD4 or to undergo morphological differentiation into the ST, in contrast to the untreated control (Fig. 1E-G, Fig. S10). Similarly, BBinh treatment impaired the ST in trophoblast organoids and prevented ST differentiation of hTSCs under the 3D protocol (Fig. S2A-E, Fig. S10). Together, our findings demonstrate that SWI/SNF activity is vital for developing the ST lineage of the human placenta.

In general, ST differentiation involves both molecular and morphological changes. While molecular changes include repression of the cell cycle and CT progenitor genes, and upregulation of genes associated with hormone production, metabolism and nutrient transport, the morphological changes comprise cytoskeletal remodelling and cell fusion. To investigate how the transcriptional impact of SWI/SNF during CT to ST differentiation unfolds over time, we followed the effects of treatment with or without BBinh after 12, 24 and 72 h of ST differentiation by 3′mRNA-seq. The principal component analysis (PCA) revealed that, although during the first 12 h the CTRL_ST and BBinh_ST followed similar differentiation dynamics, after 24 h the BBinh_ST lagged on PC1 and clustered away from the CTRL_ST. After 72 h, this effect was even more pronounced, as BBinh_ST deviated farther away from the CTRL_ST (Fig. 1H, Table S1). The top differentially expressed genes along PC1 include those associated with cell fusion (*ERVFRD-1*, *ERVW-1*, *MFSD2A* and *HSD11B2*), hormone production (*LEP*, *CYP11A1*, *CYP19A1*, *CGA* and *INSL4*) and the cytoskeleton (*TUBA4A* and *KRT23*), while along PC2 those associated with the transport function of the ST (*SLC2A1*, *SLC1A2*, *SLC20A1*, *SLC29A3* and *SLC6A6*). (Fig. 1I) To gain a better understanding of how BBinh impacts the transcriptional changes during the ST differentiation, we first identified the top 3000 variance genes. Hierarchical clustering segregated them into six clusters reflecting their distinct dynamic regulation. While cluster 2 contained the early drivers of ST identity, including TFs (e.g. *GCM1*, *TBX3* and *HOPX*) and genes necessary for syncytialization (e.g. *MFSD2A* and *ERVW-1*), cluster 1 harboured genes associated with endocrinally active ST producing hormones (e.g. *LEP*, *CGA*, and members of the CGB and PSG gene families) (Fig. 1J, Table S1). Accordingly, GO analysis showed enrichment of cluster 2 in terms related to WNT signalling, actin filament organization and cell adhesion, and enrichment of cluster 1 in terms ranging from endoplasmic reticulum to Golgi vesicle-mediated transport (Fig. S1H). Importantly, BBinh resulted in the impaired induction and/or upregulation of genes in these two clusters, indicating an early differentiation defect (Fig. 1J, Table S1). Clusters 3 and 4 represented genes that are downregulated during ST differentiation and contained stemness (e.g. *TEAD4*, *YAP1*, *MSX2* and *SP6*), and proliferation markers (e.g. *MKI67* and *CCND1*) (Fig. 1J, Table S1). They were enriched in terms related to WNT signalling, nuclear division and actin organization (Fig. S1H). Upon BBinh, these genes failed to be silenced and continued to be expressed. Genes in clusters 5 and 6 were also dysregulated, following the general trend (Fig. 1J, Table S1). Overall, these observations indicate that BBinh-treated cells failed to exit self-renewal and commit to the ST programme.

### Misregulation of the cell cycle and cyclin D1 contributes to ST differentiation failure

**Fig. 2.**
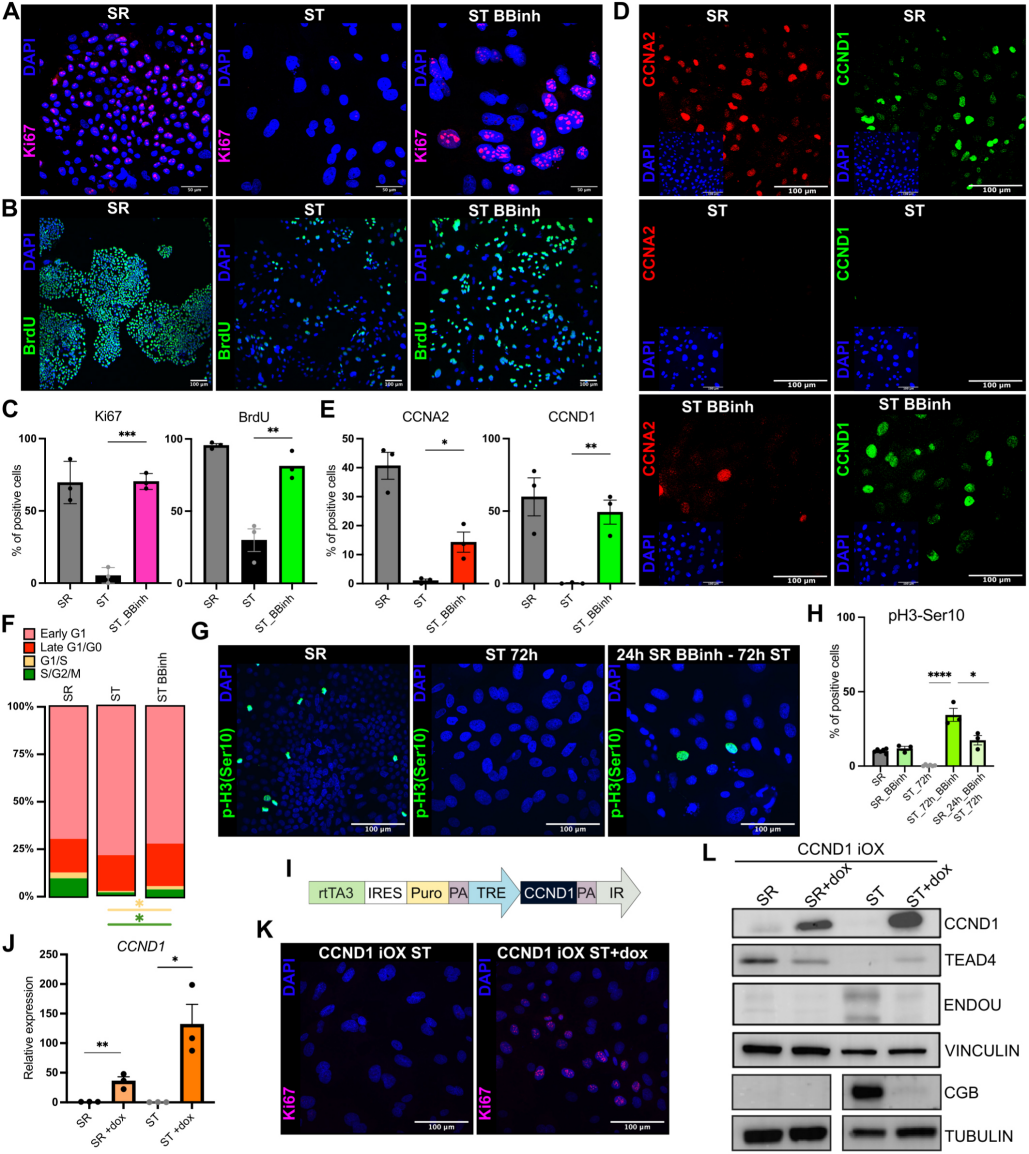
**Cell cycle and CCND1 misregulation contribute to syncytiotrophoblast differentiation failure.** (A) Immunofluorescence staining of Ki67 (magenta) and DAPI (blue) in hTSCs (SR) and syncytiotrophoblast (ST), and BBinh-treated ST at 72 h. (B) Immunofluorescence staining of BrdU (green) and DAPI (blue) in hTSCs (SR) and ST, and BBinh-treated ST at 72 h. BrdU incorporation was performed for 24 h before harvest. (C) Quantification of Ki67 and BrdU immunofluorescence signals from A and B. *P<0.05, **P<0.01, ***P<0.001, unpaired *t*-test (data are mean±s.e.m.), *n*=3, 3 areas. (D) Immunofluorescence staining of CCNA2 (red), CCND1 (green) and DAPI (blue) in hTSCs (SR), and ST and BBinh-treated ST at 72 h. (E) Quantification of CCNA2 and CCND1 immunofluorescence signals from D. *P<0.05, **P<0.01, unpaired *t*-test (data are mean±s.e.m.), *n*=3, 3 areas. (F) Flow cytometry analysis of a fluorescence ubiquitin cell cycle indicator (FUCCI) hTSC cell line in SR, ST and BBinh-treated ST at 72 h, *n*=2 (unpaired *t*-test). The data indicate the percentage of cells in each indicated cell cycle phase (early G1, late G1/G0, G1/S and S/G2/M), based on FACS gating. The graph uses a ‘Parts of Whole’ representation to show the proportion of each mean value relative to the total cell population. (G) Immunofluorescence staining of p-Histone H3 Ser10 [pH3(Ser10)] (green) and DAPI (blue) in cells cultured in SR, 72 h ST and pre-treated with BBinh for 24 h in SR followed by 72 h ST in the absence of BBinh (24h_SR_BBinh-72hST). (H) Quantification of p-H3(Ser10) immunofluorescence signals from G (SR, ST_72h and 24h_SR_BBinh-72hST), as well as cells cultured in ST_72h_BBinh and SR_BBinh. *P<0.05, ****P<0.0001, unpaired *t*-test (data are mean±s.e.m.), *n*=3, 3 areas. (I) Scheme of the CCND1 doxycycline (dox) inducible overexpression (iOX) construct. (J) RT-qPCR analysis of hTSCs with CCND1 iOX construct in SR and ST with or without dox at 72 h, *n*=3 (unpaired *t*-test, data are mean±s.e.m.). *P<0.05, **P<0.01. (K) Immunofluorescence staining of Ki67 (magenta) and DAPI (blue) in CCND1 iOX line ST treated with or without dox at 72 h. (L) Western blot of CCND1 iOX line SR and ST treated with or without dox at 72 h. Vinculin and tubulin serve as loading controls.

The misexpression of the cell cycle-related genes prompted us to investigate further how BBinh affects the cell cycle and, consequently, ST differentiation. The SWI/SNF complex has been shown to play distinct roles at various stages of the cell cycle in a context-dependent manner (Dykhuizen et al., 2013; Marquez et al., 2014). Moreover, previous studies indicate that exit from the cell cycle at G1 (G0) is a prerequisite for the formation of a stable ST (Lu et al., 2017). To validate these findings in the hTSC model, we differentiated the cells to ST in the presence or absence of BBinh for 72 h and followed Ki67 staining and bromodeoxyuridine (BrdU) incorporation. Ki67 marks the active phases of the cell cycle (G1, S, G2 and M) and it is lost in post-mitotic G0 cells (Yerushalmi et al., 2010). While the Ki67 signal was lost in control ST cells, it was readily detectable in BBinh-treated cells (Fig. 2A-C). To assess DNA synthesis, we evaluated the incorporation of BrdU, a thymidine analogue, during ST differentiation with or without BBinh. The BrdU signal was strongly reduced in ST control cells compared to the cycling self-renewing hTSCs, confirming cell cycle exit upon differentiation (Fig. 2B,C). In contrast, BBinh-treated cells during ST differentiation maintained high BrdU levels, indicating continued DNA replication and a failure to exit the cell cycle (Fig. 2B,C). Gene expression analysis further supported this observation. BBinh-treated ST cells showed increased levels of cell cycle-promoting genes *CCNA2*, *CCNB1* and *CCND1*, and reduced levels of inhibiting *P21* (*CDKN1A*) (Fig. 2D,E, and Fig. S3A). These data reinforce the role of SWI/SNF in repressing G1 progression (Marquez et al., 2014), and suggest that BBinh treatment enables progression through the G1/S, thereby preventing the G1/G0 exit necessary for proper ST differentiation (Lu et al., 2017).

To further corroborate our findings, we generated a Fluorescence Ubiquitin-based Cell Cycle Indicator (FUCCI) hTSC reporter line. The FUCCI construct has a green fluorescence protein (GFP) fused to GMNN (geminin), which is expressed during the S, G2 and M phases of the cell cycle, and a red fluorescent protein (RFP) fused to CDT1 (Cdc10-dependent transcript 1 – a DNA replication factor), expressed during late G1 and G0 phases of the cell cycle. The early G0 phase is characterized by GMNN^low^ and CDT1^low^, late G1/G0 is characterized by GMNN^low^ and CDT1^high^, G1/S transition is marked by co-expression of both GMNN and CDT1 (GMNN^high^ and CDT1^high^), and S/G2/M is characterized by GMNN^high^ and CDT1^low^ expression (Sakaue-Sawano et al., 2008). We cultured the FUCCI-hTSC line in ST conditions for 72 h in the presence or absence of BBinh. Our observations indicated that a proportion of BBinh-treated ST cells was unable to exit the cell cycle and continued cycling, as demonstrated by an increased proportion of cells in the G1/S and S/G2/M phases (Fig. 2F and Fig. S3B). Thus, SWI/SNF controls a key aspect of ST differentiation: the exit from the cell cycle.

Next, we asked how pre-treatment with the BBinh in SR, followed by 72 h of ST differentiation (without BBinh), would affect ST differentiation. We assessed phospho-Histone H3-Ser10 (pH3-Ser10), a marker of chromatid condensation during mitosis (G2/M). In control 72 h ST cells, pH3-Ser10 staining was absent, consistent with successful exit from G1/G0. In contrast, BBinh-treated ST displayed abnormally elevated pH3-Ser10 levels, indicating an accumulation in G2/M (Fig. 2G,H). Notably, a 24 h BBinh pre-treatment in SR, followed by 72 h of ST differentiation without BBinh, led to intermediate pH3-Ser10 levels (Fig. 2G,H). Similarly, expression of ST markers showed intermediate levels (Fig. S3C). These observations suggest that while cells stuck in G2/M during SR failed to differentiate, cells in later phases of the cell cycle did differentiate. They align with previous reports of the role of SWI/SNF in facilitating G2/M progression (Dykhuizen et al., 2013), in addition to repressing G1/S progression (Marquez et al., 2014).

Among the most upregulated genes following BBinh treatment was CCND1, a key driver of G1/S progression via inactivation of pRB and activation of genes required for DNA replication (Klein and Assoian, 2008). To explore its potential role in mediating the BBinh phenotype, we generated a doxycycline (dox)- inducible *CCND1* overexpression (iOX) hTSC line (Fig. 2I,J and Fig. S3D). Upon simultaneous induction of *CCND1* and ST differentiation for 72 h, we observed impaired differentiation, marked by persistent Ki67 positivity, prolonged TEAD4 expression, and attenuated induction of ST markers CGB and ENDOU (Fig. 2K,L, Fig. S10 and Fig. S3D-F). To test whether CCND1 downregulation could rescue the BBinh-induced differentiation defects, we co-treated cells with BBinh and CCND1 shRNA constructs (KD1 and KD2). Although both constructs effectively reduced CCDN1 expression and partially restored ST marker expression, they failed to fully reverse the effects of BBinh (Fig. S3G). This suggests that while CCND1 misregulation contributes to the impaired differentiation, it is not the sole driver of the phenotype. In conclusion, our findings demonstrate that BBinh treatment disrupts ST differentiation by altering cell cycle dynamics, with differential effects depending on the cell phase at the time of exposure. Cells in G1 phase fail to exit the cycle due to accelerated progression through G1/S, consistent with the role of SWI/SNF in repressing G1 progression (Marquez et al., 2014). Conversely, cells in S/G2 accumulate at the G2/M transition, reflecting a requirement for SWI/SNF in facilitating G2/M progression (Dykhuizen et al., 2013). Thus, SWI/SNF activity is essential for proper cell cycle regulation and exit, and successful ST differentiation.

### Inhibition of BRG1 and BRM function attenuates chromatin dynamics during ST differentiation

**Fig. 3.**
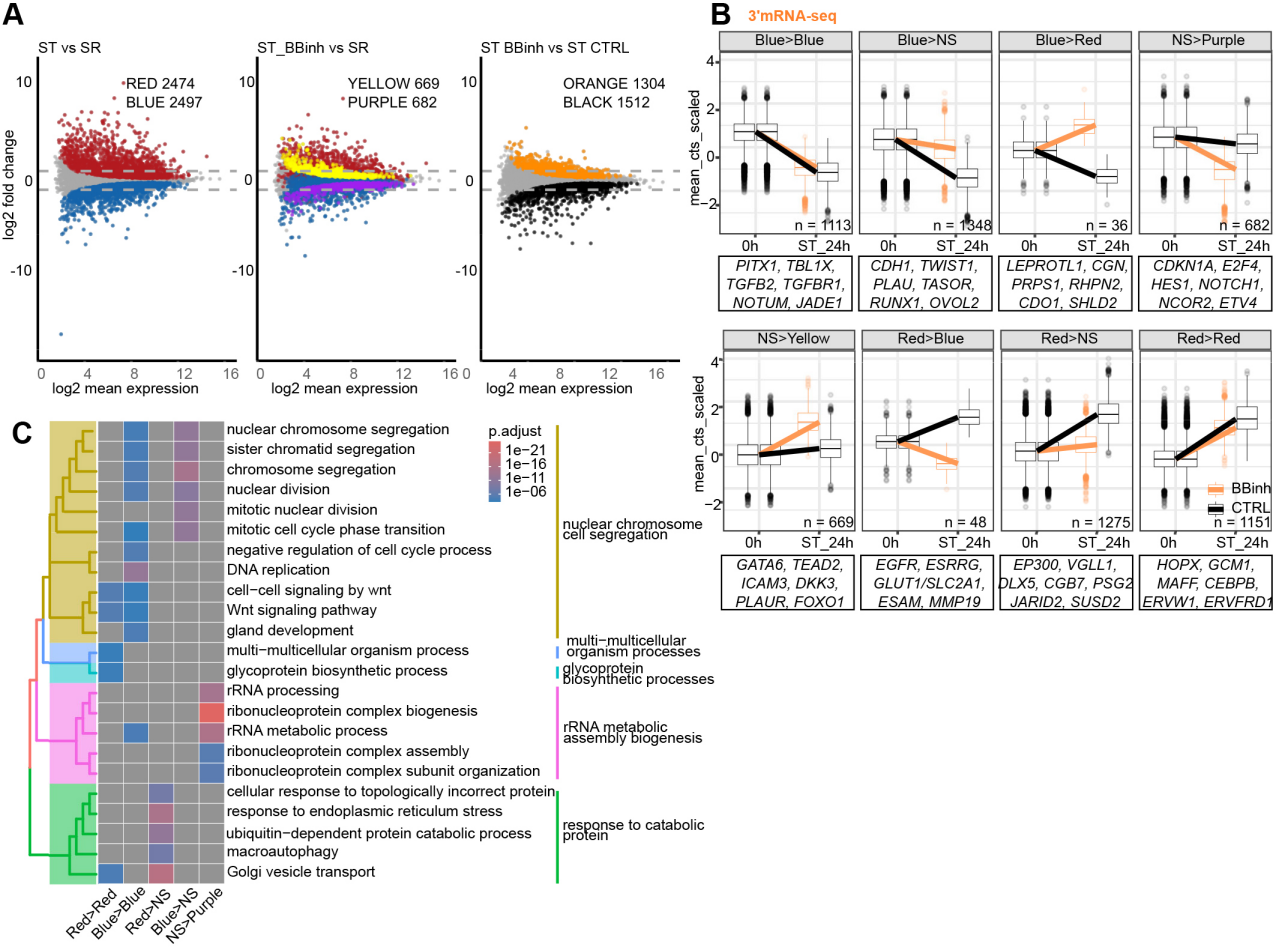
**Inhibition of BRG1/BRM function attenuates gene expression profile changes during syncytiotrophoblast differentiation.** (A) MA plots showing how differentially expressed genes in syncytiotrophoblast (ST) versus self-renewing (SR) cells (24 h; *n*=3, cutoff: *P* adj<0.05) in blue (downregulated) and red (upregulated) behave during differentiation from SR to ST BBinh (ST BBinh 24 h versus ST CTRL 24 h). Genes that are significantly regulated in ST BBinh versus SR only (24 h; *n*=3, cutoff: *P* adj<0.05) are shown in purple (downregulated) and yellow (upregulated). Differentially expressed genes in ST BBinh versus ST CTRL are shown in orange (upregulated in ST BBinh) and black (downregulated in ST BBinh). (B) Line plots of aggregated gene expression (scaled vst counts) of group transitions in the comparison of ST versus SR and ST BBinh versus SR from (A) (B, blue; R, red; Y, yellow; P, purple; NS, non-significant). Box boundaries show the 25th to 75th percentile, with the median as the centre and whiskers representing the calculated maximum and minimum. Outliers are depicted by the dots, *P*_adj<0.05 (NS, non-significant). (C) Tree plot of GO term enrichment of groups identified in A (NS, non-significant).

Having established the crucial role of SWI/SNF in ST differentiation, we next sought to more precisely investigate its timing and the underlying molecular mechanisms. Since our PCA and cluster analysis demonstrated that the first pronounced transcriptional changes manifest after 24 h of ST differentiation regardless of the BBinh treatment, we focused on the dynamics of the differentiation at this early time point. After 24 h of the SR to ST differentiation, we observed massive transcriptional changes, with 2474 genes being significantly upregulated and 2497 genes downregulated (Fig. 3A left), reflecting a major shift. Next, we asked how these genes behaved in the presence of BBinh during SR to ST_BBinh. Our analysis revealed that while 1151 upregulated (Red>Red) and 1113 downregulated (Blue>Blue) genes maintained differential expression upon BBinh treatment, a large proportion was no longer significantly regulated (Red>NS and Blue>NS) or even conversely regulated (Blue>Red and Red>Blue) compared to the untreated control (Fig. 3A,B). These genes were enriched in GO terms related to response to catabolic protein and nuclear chromosome and sister chromatid segregation, respectively (Fig. 3B,C). Moreover, 669 genes became specifically induced and 682 repressed upon BBinh treatment (Fig. 3A,B). Finally, direct comparison of the ST_BBinh and ST conditions showed a large number of differentially expressed genes. Downregulated genes (1512) included regulators of ST identity, whereas upregulated (1304) included cell cycle regulators (Fig. 3A right). Taken together, these results suggest that the SWI/SNF complex is a key regulator of ST differentiation in the first 24 h, as it controls the expression of a large number of genes, including crucial G1/S and G2/M cell cycle regulators.

**Fig. 4.**
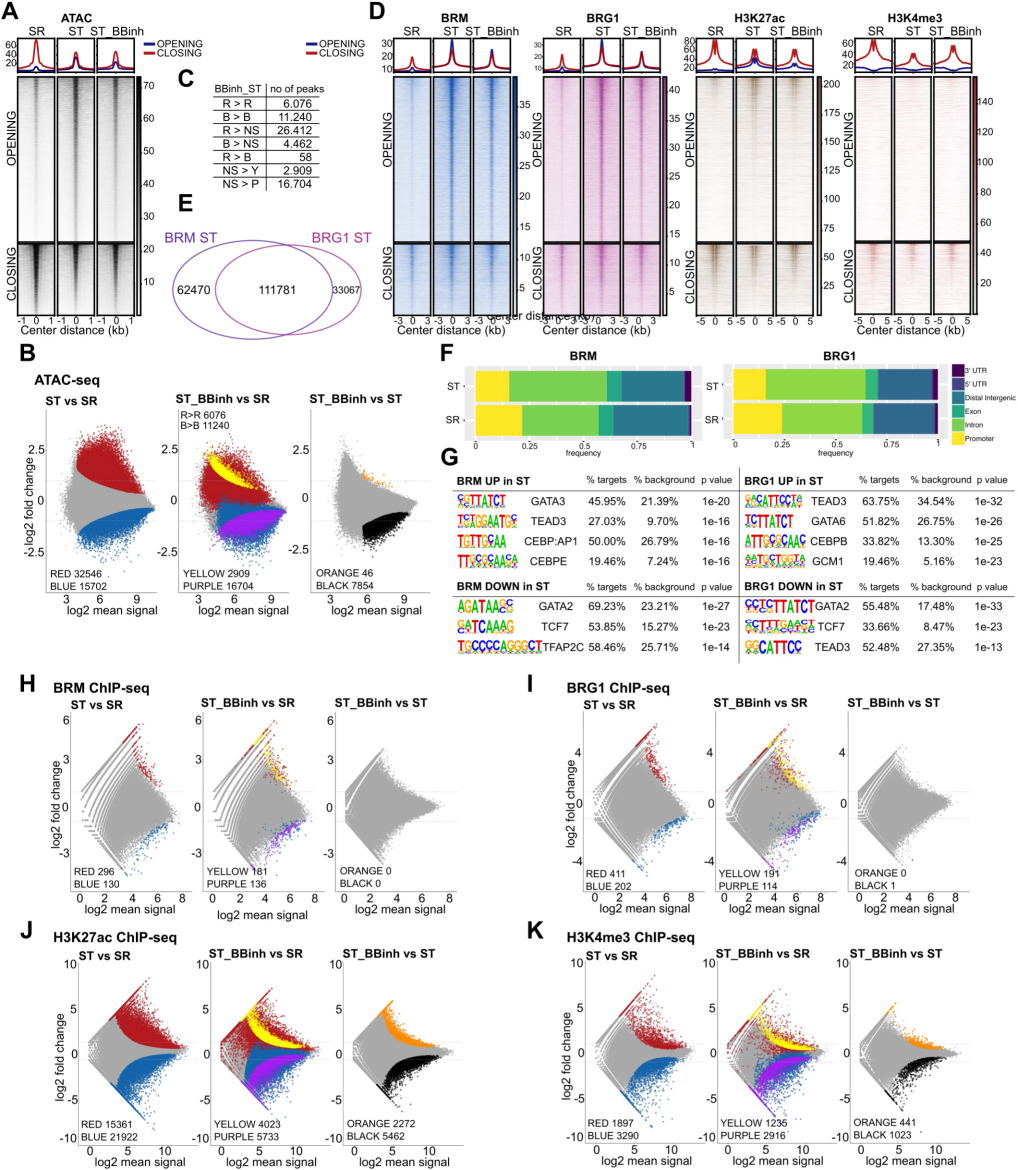
**Inhibition of BRG1/BRM function attenuates chromatin architecture during syncytiotrophoblast differentiation.** (A) Heatmap showing ATAC-seq signal in ‘OPENING’ and ‘CLOSING’ regions, as determined by differential accessibility analysis of ATAC-seq peaks in untreated 24 h syncytiotrophoblast (ST) versus self-renewing (SR) hTSCs. (B) MA plots showing how differentially opened regions in ST versus SR hTSCs, as determined by ATACseq (24 h; *n*=3, cutoff: *P* adj<0.05) in blue (downregulated) and red (upregulated), behave in the transition from SR to ST BBinh (ST BBinh 24 h versus ST CTRL 24 h). Only regions that are significantly regulated in ST BBinh (24 h; *n*=3, cutoff: *P* adj<0.05) are shown in purple (downregulated) and yellow (upregulated). Differentially opened regions in ST BBinh versus ST CTRL are shown in orange (upregulated in ST BBinh) and black (downregulated in ST BBinh). (C) The number of ATAC-seq regions identified in B in the transition ST versus SR to ST_BBinh versus SR (B, blue; R, red; Y, yellow; P, purple; NS, non-significant). R>R, regions that remained open; B>B, regions that remained closed; R>NS, regions that failed to open; B>NS, regions that failed to close; Y, regions that specifically opened upon BBinh; P, regions that specifically closed upon BBinh; R>B, regions that transitioned from open to closed. (D) Heatmap of BRM (blue), BRG1 (magenta), H3K27ac (brown) and H3K4me3 (red) ChIP-seq signal in ‘OPENING’ and ‘CLOSING’ regions of A. (E) Venn diagram showing the overlap between regions bound by BRM and BRG1 in ST. (F) Feature distribution of ATAC-seq regions differentially bound by BRM and BRG1 in ST versus SR, as determined by ChIP-seq and DiffBind (see the red and blue groups in H and I). (G) *De novo* motifs identified by HOMER on ATAC-seq regions differentially bound by BRM and BRG1. (H) MA plots showing BRM enrichment on ATAC-seq consensus peaks (found in ST and SR). (I) MA plots showing BRG1 enrichment on ATAC-seq consensus peaks (from A). (J) MA plots showing H3K27ac enrichment in ATAC-seq consensus peaks (from A). (K) MA plots showing H3K4me3 enrichment in ATAC-seq consensus peaks (from A). For all MA plots, differentially enriched regions in ST (24 h) versus SR (*n*=3, cutoff: *P* adj<0.05; left) are shown in blue (downregulated) and red (upregulated). Regions that are only significantly regulated in ST BBinh (*n*=3, cutoff: *P* adj<0.05, right) are shown in purple (downregulated) and yellow (upregulated). Differentially enriched regions in ST BBinh versus ST are shown in orange (upregulated in ST BBinh) and black (downregulated in ST BBinh).

As BBinh inhibits the ATPase activity of BRM and BRG1, we sought to determine changes in chromatin accessibility during 24 h of ST differentiation in the presence or absence of BBinh using the Assay for Transposase-Accessible Chromatin sequencing (ATAC-seq). We first defined the set of accessible regions by merging ATAC-seq peaks from both the SR and ST conditions. We then looked at the chromatin dynamics during the 24 h SR to ST specification using differential accessibility analysis and identified 32,456 opening (red, R) and 15,702 closing (blue, B) regions, reflecting the cell identity shifts (Fig. 4A-C, Table S2). Next, we asked whether these regions were affected by the BBinh. While 6076 opening and 11,240 closing regions maintained their differential accessibility (R>R and B>B), a substantial proportion failed to open (26,412; R>NS) or close (4462; B>NS) upon BBinh treatment (Fig. 4A-C, Table S2). GO enrichment analysis of the 6076 regions that remained open despite BBinh treatment (R>R) revealed significant enrichment for pathways related to GTPase-mediated signal transduction, cell junction assembly and Wnt signalling – key pathways associated with hTSCs and CT function (Fig. S4A). Notably, 2909 regions specifically opened (NS>Y, yellow) and 16,704 closed (NS>*P*, purple) upon BBinh treatment, highlighting the crucial role of BRG/BRM in promoting chromatin opening (Fig. 4A-C, Table S2). A direct comparison between ST_BBinh and untreated ST revealed only 46 regions that specifically opened in the presence of BBinh, in contrast to 7854 that remained closed. This further underscores the essential function of the SWI/SNF complex in driving chromatin accessibility within the first 24 h of ST differentiation (Fig. 4A-C, Table S2).

Next, to assess whether BRG1/BRM occupancy changes during the SR to ST differentiation, we performed chromatin immunoprecipitation (ChIP-seq) (Fig. 4D and Fig. S4B-E). We focused our analysis on the previously defined accessible regions (ATAC-seq peaks) (Fig. 4D, Table S3). BRG1 and BRM exhibited substantial overlap in their binding profiles (Fig. 4E and Fig. S4B). Genomic feature analysis revealed that both factors primarily occupied non-promoter regions, although promoter binding was more prominent in SR than ST (Fig. 4F and Fig. S4C). The differential binding analysis uncovered 130 BRM and 202 BRG1 regions with increased binding in SR conditions. These regions were enriched in GATA2-, TCF7-, TEAD3- and TFAP2C-binding motifs (Fig. 4G-I, Table S3), in line with the known roles of the corresponding TFs in driving hTSC stemness and self-renewal (Chen et al., 2022; Dong et al., 2022; Ghosh et al., 2024; Papuchova and Latos, 2022; Saha et al., 2020). The analysis also identified 296 and 411 regions with increased binding of BRM and BRG1, respectively, after 24 h of ST differentiation (Fig. 4H,I, Table S3). In addition to TEAD3 and GATA3, they were enriched in CEBPB, CEBPE and GCM1 binding motifs, and both GCM1 and Cebpa/Cebpb TFs are essential drivers of ST differentiation (Jeyarajah et al., 2022; Simmons et al., 2008; Zhu et al., 2023) (Fig. 4G). Upon BBinh during ST differentiation, some regions showed differential enrichment of BRM and BRG1 only in ST BBinh (yellow and purple). A direct comparison of ST_BBinh with ST revealed neither BRM nor BRG1 differentially bound regions (Fig. 4F,G, Table S3). Together, these observations indicate that the SWI/SNF complex binding exhibits a low degree of dynamism during the SR to ST differentiation, and defective SWI/SNF binding does not cause the ST differentiation blockage observed upon BBinh.

**Fig. 5.**
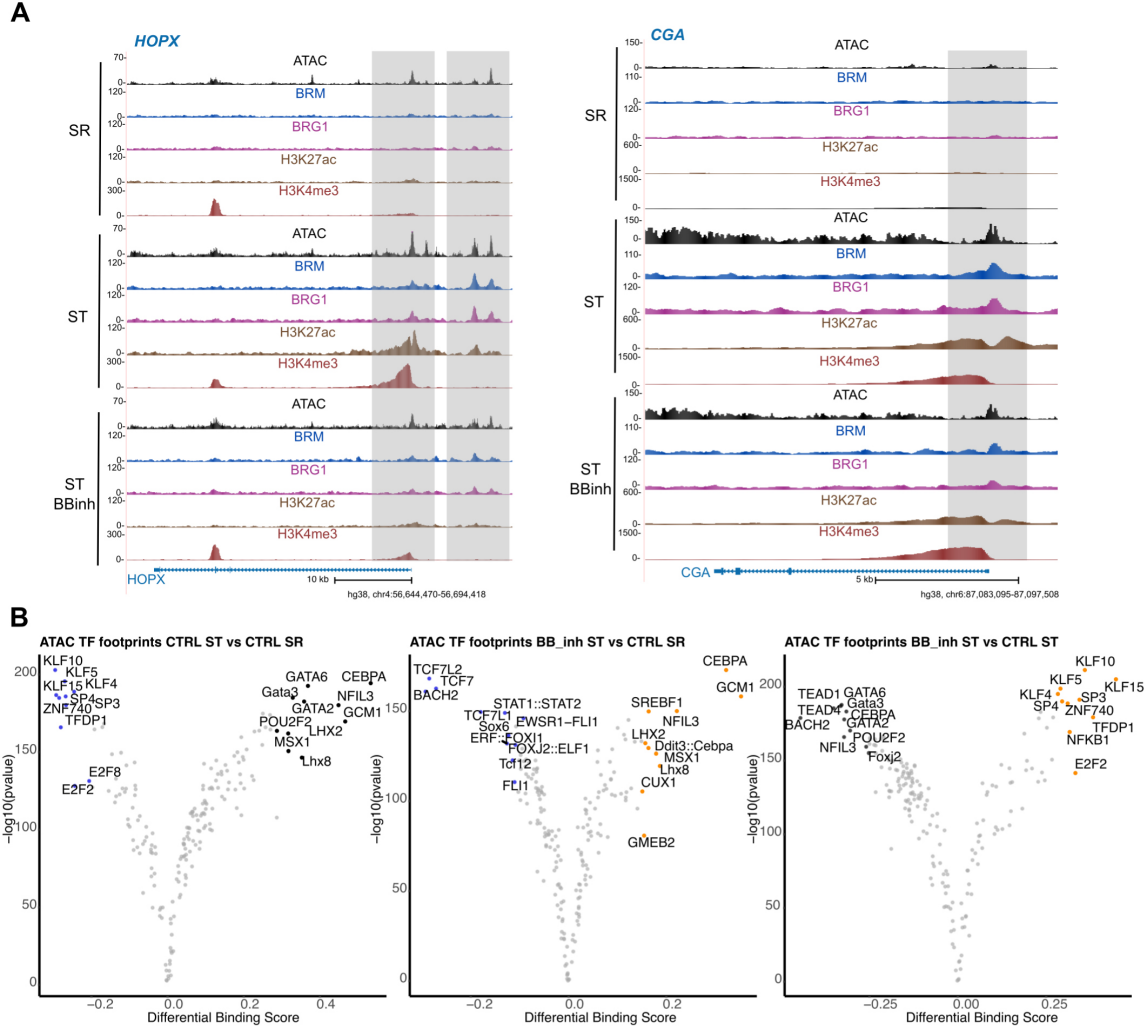
**Identification of SWI/SNF-dependent regulators of syncytiotrophoblast differentiation.** (A) Signal profile plots of the heatmaps from Fig. 4A and signal profile plots of Fig. 4D showing ChIP-seq on HOPX and CGA gene regions. (B) Volcano plots demonstrating the differential binding of TFs, as predicted by TOBIAS between control SR (blue) versus syncytiotrophoblast (ST) (black, left panel) and BBinh-treated ST (orange) and control ST (black, right panel).

Alterations in chromatin accessibility are associated with dynamic changes in histone modifications. Open chromatin regions often accumulate H3K4me3 and H3K27ac, and are linked to promoters and gene regulatory elements, respectively (Hnisz et al., 2013; Shlyueva et al., 2014; Thomas and Buecker, 2023). Thus, we followed H3K4me3 and H3K27ac enrichment during the 24 h SR to ST differentiation in the presence and absence of BBinh. In line with previous reports (Basurto-Cayuela et al., 2024; Iurlaro et al., 2021; Schick et al., 2021), we observed that changes in chromatin accessibility were accompanied by dynamic changes in histone modifications (Fig. 4J,K, Table S3). Interestingly, we observed more prominent changes in H3K27ac compared to H3K4me3, indicating that SWI/SNF predominantly regulates enhancers. Among the affected genes, we found the key ST markers (Figs 4J,K, and 5A). Next, we focused our analysis on promoter regions that failed to open during ST_BBinh differentiation (R>NS+R>B, Fig. 4B middle) and simultaneously did not acquire enrichment for H3K27ac (R>NS+R>B, Fig. 4J middle) and H3K4me3 (R>NS+R>B, Fig. 4K middle) histone marks (Fig. S4F). We first identified 2441 genes linked to promoter regions that remained inaccessible and lacked H3K27ac, which were significantly enriched for terms related to the placenta and ST (Fig. S4F,G). Further narrowing the analysis to promoter regions that also failed to gain H3K4me3 revealed a smaller set of 52 genes, still enriched for placental and ST function. Notably, this group included *GCM1*, a well-established master regulator of trophoblast differentiation (Anson-Cartwright et al., 2000; Jeyarajah et al., 2022; Shimizu et al., 2023), alongside other known ST markers (Fig. S4F,H). These findings suggest that a subset of ST genes, including key regulators like GCM1, require coordinated chromatin opening and histone modification to become transcriptionally competent during ST differentiation. Their failure to do so under BBinh highlights a potential epigenetic blockade that is crucial to impaired ST development.

**Fig. 6.**
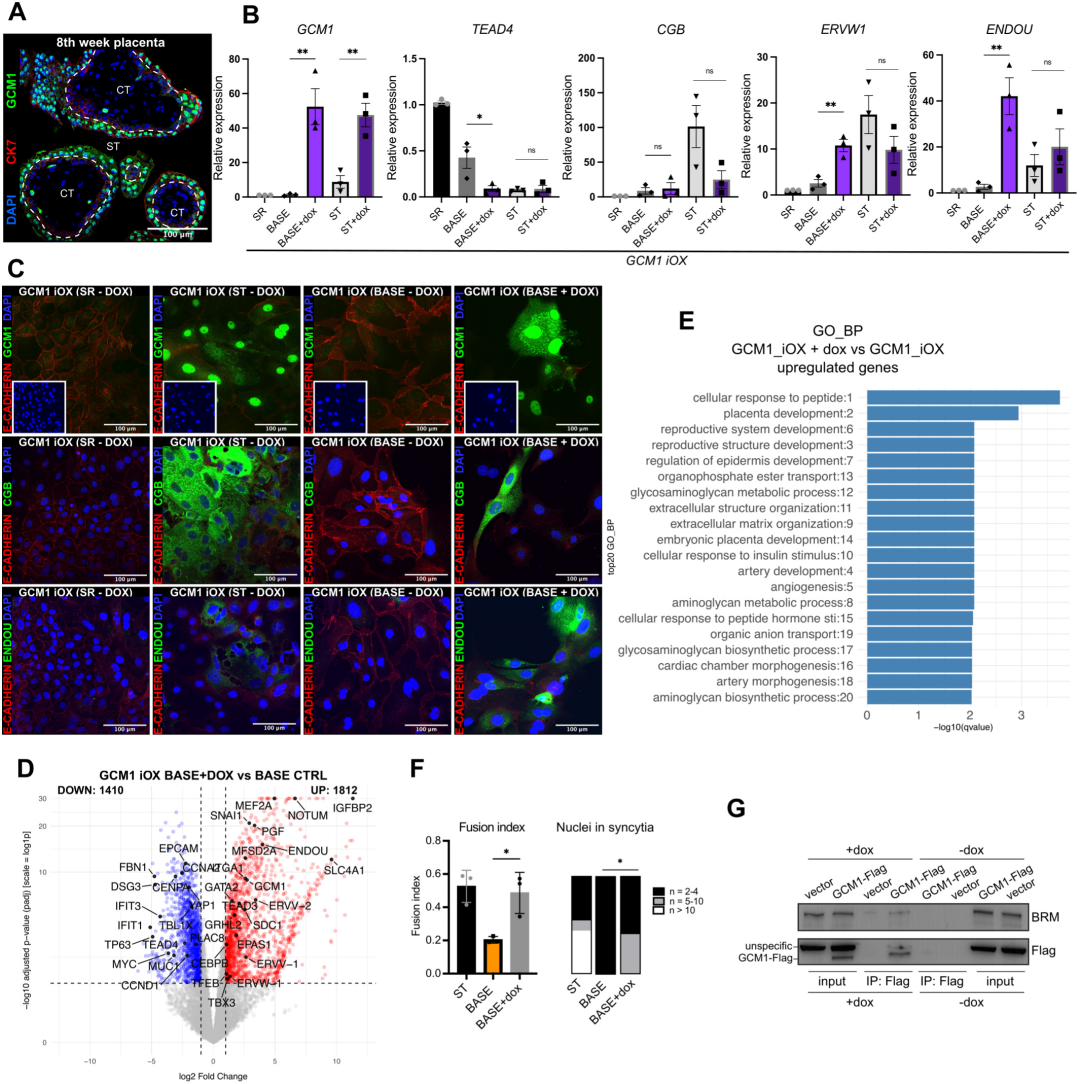
**GCM1 is a major driver of the syncytiotrophoblast transcriptional program.** (A) Immunofluorescence staining of human placental tissue (8th week) for GCM1 (green), CK7 (red) and DAPI (blue). (B) RT-qPCR analysis of hTSCs with GCM1 iOX construct in self-renewing (SR) cells, BASE, BASE+dox and syncytiotrophoblast (ST) at 72 h, *n*=3 *P<0.05, **P<0.01 (unpaired *t*-test, data are mean±s.e.m.). (C) Immunofluorescence staining of hTSCs with the GCM1 iOX construct in self-renewing (SR), ST and BASE (all without dox), and induced GCM1 iOX in BASE+dox at 72 h. Top: GCM1 (green), E-cadherin (red) and DAPI (blue). Middle: CGB (green), E-cadherin (red) and DAPI (blue). Bottom: ENDOU (green), E-cadherin (red) and DAPI (blue). (D) Volcano plot from differentially expressed genes between GCM1 iOX Base+dox and GCM1 iOX BASE-dox (CTRL) (*n*=3, cutoff: |log2FC|>1, *P* adj<0.05). (E) Gene Ontology (GO) biological process (BP) enrichment of genes upregulated in GCM1 iOX in BASE+dox versus BASE control, (*n*=3, cutoff: |log2FC|>1, *P* adj<0.01). (F) Quantification of GCM1 iOX fusion index [fusion index=(number of nuclei in syncytia−number of syncytia)/total number of nuclei]. Syncytium was defined by two or more nuclei. *P<0.05, unpaired *t*-test (data are mean±s.e.m.), *n*=3, 3 areas, 2 independent observers, 72 h. (G) Anti-Flag co-immunoprecipitation in ST cells expressing dox-inducible GCM1-Flag iOX or empty vector iOX control, analysed by western blot with indicated antibodies.

In addition to providing insights into chromatin accessibility, ATAC-seq can be used to explore TF binding. A bound TF protects the DNA from the transposase (Tn5) activity, causing a dip in the ATAC-seq signal at a given motif. This allows for predictions of differential binding (protected from Tn5 activity) or ejection (the binding site is exposed to Tn5) of TFs. We used TOBIAS (Bentsen et al., 2020) to identify the TF DNA-binding motifs specific for SR, ST and BBinh_ST states. The footprinting analysis of SR to ST differentiation revealed distinct sets of TF-binding motifs associated with each cell state. In the SR state, motifs enriched included members of the KLF (KLF10, KLF5, KLF4 and KLF15), SP (SP3 and SP4) and E2F (E2F8 and E2F2) TF families. (Fig. 5B). Consistent with this, several of these TFs are expressed in CT and hTSCs, where they have been implicated in maintaining the progenitor state (Chen et al., 2024; Dou et al., 2024; Ishiuchi et al., 2019). In contrast, ST-enriched motifs included members of the GATA (GATA1, GATA2 and GATA3) and LHX (LHX2 and LHX8) families, as well as CEBPA, MSX1, GCM1 and POU2F2. These findings are in agreement with the increased expression of these TFs in ST and their established roles in promoting ST identity (Anson-Cartwright et al., 2000; Ghosh et al., 2024; Jeyarajah et al., 2022; Ounadjela et al., 2024) (Fig. 5B). Interestingly, the BBinh-treated ST displayed enrichment of motifs typically associated with the SR state, including KLF4, KLF5, KLF10, KLF15, SP3, SP4, ZNF740, NFKB1 and E2F2. This motif profile correlates with the impaired ST differentiation phenotype observed upon BBinh treatment (Fig. 5B). Given that BBinh disrupts chromatin accessibility, we propose that this results in reduced binding of ST-specifying TFs due to a more inaccessible chromatin landscape, leading to the persistence of SR-specific factors. This analysis provides a foundation for future functional studies aimed at dissecting the regulatory networks of these TFs. Collectively, these findings highlight the crucial role of chromatin remodelling in enabling TF function during hTSC maintenance and differentiation.

### GCM1 is a major driver of the ST program

Our HOMER (Fig. 4G) and TOBIAS (Fig. 5B) analysis implicated GCM1 and CEBPA/CEBPB, among other TFs, as the SWI/SNF-associated early regulators of ST identity. Studies in mutant mice have demonstrated that *Gcm1* and *Cebpa* are essential drivers of the ST layer II (SynT-II) formation (Simmons et al., 2008). Forced expression of *Gcm1* in murine TSCs resulted in proliferation arrest and lineage restriction toward the SynT fate (Hughes et al., 2004). In humans, the GCM1 protein is also present in the ST layer of the placenta (Fig. 6A). Notably, overexpression of *GCM1* during signalling-induced ST differentiation of human TSCs enhanced the process (Jeyarajah et al., 2022), underscoring its role as a key cell fate regulator. These observations prompted us to test whether expression of *GCM1*, *CEBPB* or *CEBPA* is sufficient to drive ST differentiation in basal media lacking extrinsic signalling support. In addition, we included several of the TFs identified by the Boroviak lab as components of the early ST regulatory network – TBX3, MAFF, HOPX, NR2F6 (Chen et al., 2022) – as well as factors identified in our TOBIAS analysis: JUNB, E2F4 and FOSL1. All these factors are abundantly expressed in the ST (Chen et al., 2022; Wang et al., 2024). For each tested TF, we cloned a Flag-tagged protein-coding sequence under the control of a doxycycline (dox)-inducible promoter and generated a stable hTSC line (Fig. S5A). We cultured the cell lines in TS base media devoid of any growth factors and inhibitors (BASE conditions) in the presence and absence of dox for 72 h. Ectopic expression of *TBX3*, *MAFF*, *HOPX*, *NR2F6*, *JUNB*, *E2F4*, *FOSL1*, *CEBPA* and *CEBPB* did not result in the clear induction of ST differentiation (Figs S5B-J and S6A-J). In contrast, induced expression of *GCM1* led to the induction of ST markers (*ERVW-1*, *ENDOU* and CGB genes) in the absence of relevant signalling cues (Fig. 6B-C). Transcriptomic profiling of GCM1-iOX cells cultured under BASE conditions in the presence or absence of dox revealed significant upregulation of ST-associated genes, alongside downregulation of others, upon GCM1 induction. In addition to ST markers (e.g. *ERVW-1*, *PGF*, *ENDOU* and *MFSD2A*), several EVT-associated genes (e.g. *NOTUM*, *SNAI1* and *EPAS1*) were also upregulated, consistent with recent findings implicating GCM1 as a regulator of both ST and EVT lineages (Jeyarajah et al., 2022; Shimizu et al., 2023). (Fig. 6D and Fig. S7A, Table S1). GO analysis of the upregulated genes revealed enrichment in biological processes such as cellular response to peptide, placenta development and reproductive system development (Fig. 6E). Forced expression of GCM1 also triggered cell fusion, a hallmark of ST differentiation (Fig. 6C,F). To explore potential cooperation between GCM1 and the SWI/SNF complex, we performed co-immunoprecipitation followed by western blot and found that GCM1 interacts with BRG1 under ST conditions (Fig. 6G, Fig. S10). Further analysis of BRG1, BRM (this study) and GCM1 (Shimizu et al., 2023) ChIP-seq datasets revealed co-occupancy at a shared set of regulatory targets, including key ST genes (Fig. S7B, Table S3). These findings suggest that GCM1 may collaborate with the SWI/SNF complex to activate the transcriptional program underlying ST differentiation. To test whether GCM1 function is dependent on SWI/SNF activity, we cultured GCM1-iOX cells in BASE media with or without dox and BBinh for 72 h. Notably, inhibition of SWI/SNF abrogated GCM1-induced ST differentiation, indicating that chromatin remodelling is essential for GCM1 function and for proper activation of the ST program (Fig. S7C).

**Fig. 7.**
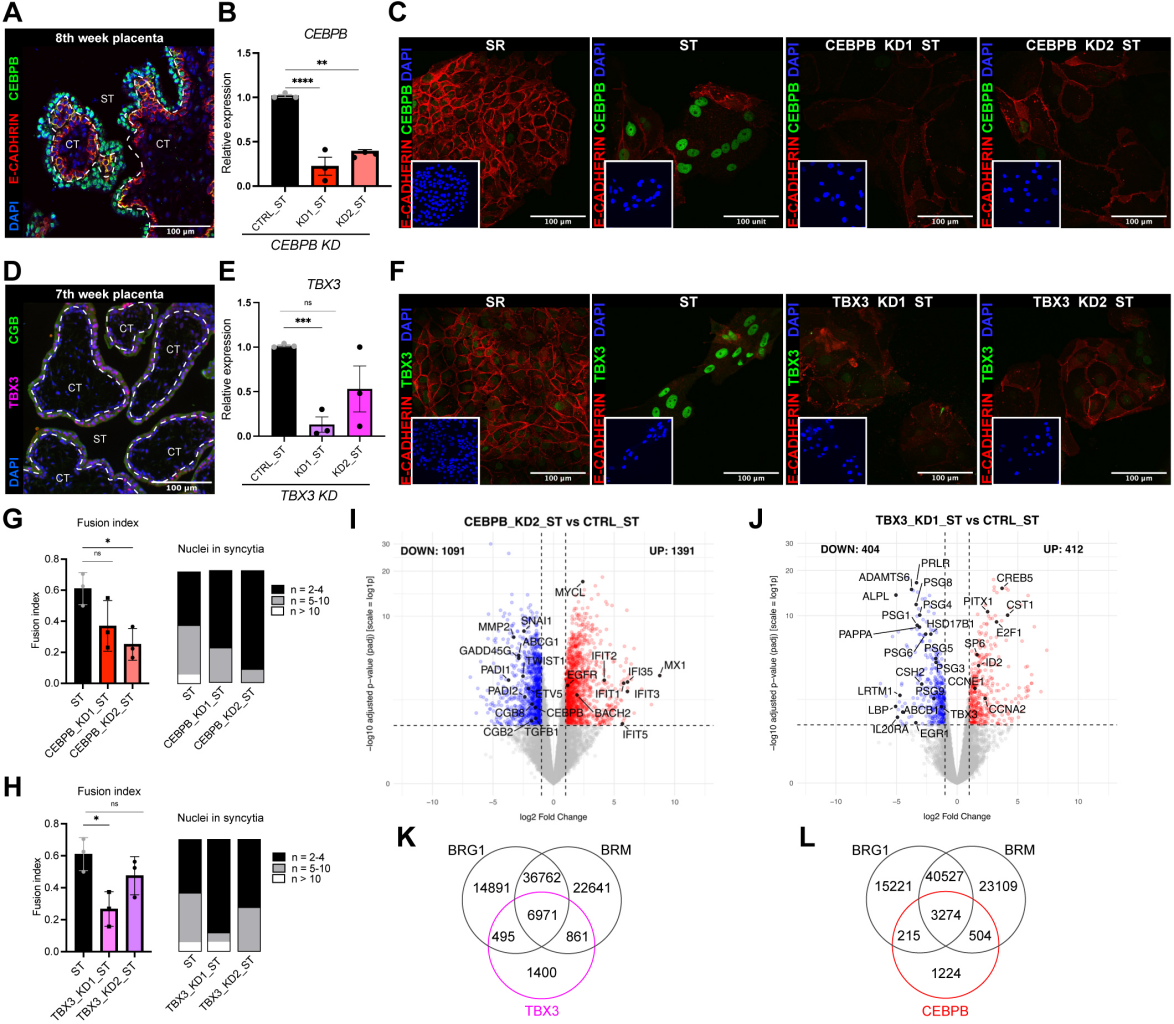
**CEBPB and TBX3 aid syncytiotrophoblast differentiation.** (A) Immunofluorescence staining of a placental section (8th week placenta) for CEBPB (green), E-cadherin (red) and DAPI staining (blue). (B) RT-qPCR analysis of *CEBPB* expression in two independent shRNA CEBPB KD lines (KD1 and KD2) and control line (CTRL) in syncytiotrophoblast (ST) at 72 h, *n*=3. **P<0.01, ****P<0.0001 (unpaired *t*-test, data are mean±s.e.m.). (C) Immunofluorescence staining of E-cadherin (red), CEBPB (green) and DAPI (blue) in self-renewing (SR) CTRL, ST CTRL and ST CEBPB KD at 72 h. (D) Immunofluorescence staining of a placental section (7th week placenta) for TBX3 (magenta), CGB (green) and DAPI staining (blue). (E) RT-qPCR analysis of *TBX3* expression in two independent shRNA *TBX3* KD lines (KD1 and KD2) and a control line (CTRL) in ST at 72 h, *n*=3.  ***P<0.001 (unpaired *t*-test, data are mean±s.e.m.). (F) Immunofluorescence staining for E-cadherin (red), TBX3 (green) and DAPI (blue) in SR, ST and TBX3 KD ST at 72 h. (G) Quantification of *CEBPB* KD fusion index [Fusion index=(number of nuclei in syncytia−number of syncytia)/total number of nuclei]. Syncytium was defined by two or more nuclei. *P<0.05, unpaired *t*-test (data are mean±s.e.m.), *n*=3, 3 areas, 2 independent observers, 72 h. (H) Quantification of *TBX3* KD fusion index [Fusion index=(number of nuclei in syncytia−number of syncytia)/total number of nuclei]. Syncytium was defined by two or more nuclei. *P<0.05, unpaired *t*-test (data are mean±s.e.m.), *n*=3, 3 areas, 2 independent observers, 72 h. (I) Volcano plot from differentially expressed genes between CEBPB_KD2_ST and ST control (72 h) (*n*=3, cutoff: |log2FC|>1, *P* adj<0.05). (J) Volcano plot from differentially expressed genes between TBX3_KD1_ST and ST control (72 h) (*n*=3). Analysis (cutoff: |log2FC|>1, *P* adj<0.05). (K) Venn diagram showing the overlap between regions bound by TBX3 and regions co-bound by BRG1 and BRM, as identified by ChIP-seq in 72 h ST and 24 h ST, respectively. (L) Venn diagram showing the overlap between regions bound by CEBPB and regions co-bound by BRG1 and BRM, as identified by ChIP-seq in 72 h ST and 24 h ST, respectively. The top 10,000 peaks of TBX3 and CEBPB were selected based on the top 25% fold enrichment and the top 75% q values.

Next, we asked which of the other identified TFs (TBX3, MAFF, HOPX, NR2F6, E2F4, JUNB, FOSL1, CEBPA, CEBPB and GCM1) are essential for ST. We depleted each TF using two different short hairpin (sh)RNAs along with a control shRNA (CTRL) by lentiviral transduction at the onset of ST differentiation of hTSC and followed the effects after 72 h (Fig. S8A, Table S4). Depletion of *MAFF*, *HOPX*, *NR2F6*, *E2F4*, *JUNB*, *FOSL1* and *CEBPA* did not seemingly affect ST differentiation, despite affecting the expression of some ST markers (Fig. S8B-H). In contrast, the abrogation of *GCM1*, *CEBPB* and *TBX3* expression hindered ST differentiation (Fig. 7A-F and Figs S7D-F and S9A,B), confirming GCM1 as a key driver of ST identity (Jeyarajah et al., 2022). Knockdown of *TBX3* (TBX3_KD1) and *CEBPB* (CEBPB_KD2) significantly reduced the fusion index, and global transcriptome analysis revealed widespread gene misregulation (Fig. 7G-J, Table S1). *TBX3* depletion led to downregulation of genes crucial for ST differentiation, including pregnancy-specific glycoproteins (*PSG1*, *PSG2*, *PSG3*, *PSG5*, *PSG6*, *PSG8*, *PSG9*), *HSD17B1*, *CSH2* and *PAPPA*, consistent with a previous report (Lv et al., 2019). *CEBPB* knockdown resulted in the upregulation of interferon signalling genes (e.g. *IFIT2*, *IFIT1*, *IFIT3*, *IFI35*, *IFIT35*, *MX1*) and downregulation of ST-associated genes such as *CGB8* and *CGB2* (Fig. 7I,J, Table S1). To identify direct transcriptional targets, we performed ChIP-seq for TBX3 and CEBPB in wild-type ST at 72 h. Comparison with genes dysregulated upon their depletion revealed a high degree of overlap for TBX3 and a lower degree for CEBPB, suggesting that TBX3 directly regulates key genes involved in ST differentiation, including *PRLR*, *PAPPA*, *SLC11A1* and the PSG family members (Fig. S9C,D, Table S3). We next compared chromatin regions bound by SWI/SNF in the ST at 24 h with those bound by TBX3 and CEBPB at in the ST at 72 h. This analysis revealed considerable overlap, suggesting potential cooperation between the SWI/SNF chromatin remodelling complex and these TFs in orchestrating ST differentiation (Fig. 7K-L). Together, our data indicate that while TBX3, CEBPB and GCM1 are necessary for ST differentiation, only GCM1 is sufficient to induce the ST programme in the absence of the relevant signalling environment. These findings – together with the enrichment of the GCM1-binding motifs in BRG1/BRM-bound regions that gain chromatin accessibility during the first 24 h of ST differentiation, the direct interaction between GCM1 and the SWI/SNF complex, and their co-occupancy of chromatin regions – strongly support that cooperation between GCM1 and SWI/SNF is essential for the induction of ST identity.

## DISCUSSION

Here, we have demonstrated that the catalytic activity of the SWI/SNF remodelling complex is essential for the differentiation from CT to ST and, thus, indispensable for human placental development and successful pregnancy outcomes. The role of the SWI/SNF complex as a vital regulator of cell fate transitions has been demonstrated in various developmental contexts. Depletion of Brg1 in oocytes resulted in the two-cell arrest and showed that Brg1 is required for the zygotic genome activation (Bultman et al., 2006), while Brg1 zygotic knockout mice die around the peri-implantation state (Bultman et al., 2000). Both mESCs and human ESCs devoid of Brg1 are unstable, fail to self-renew and display severe differentiation defects (Ho et al., 2009; Zhang et al., 2014). Brg1 was also shown to control cell fates in neural, muscle and immune lineages (Bossen et al., 2015; Lessard et al., 2007; Zhu et al., 2023). Specifically, BRG1 is required to establish chromatin accessibility at neuroectoderm-specific enhancers during hESC differentiation into neural progenitors (Hoffman et al., 2024). Loss of BRG1 in this context promoted neural crest differentiation instead. Conversely, a recent study demonstrated that Brg1 cooperates with the TF Eomes to enable mesendoderm enhancer accessibility and, thereby, differentiation (Schröder et al., 2025). While the role of SWI/SNF during early embryonic specification events was explored, its function in trophoblast differentiation remained obscure. Thus, our findings that inhibition of SWI/SNF abrogates ST differentiation are in line with its role as a major developmental regulator of embryonic and extraembryonic lineages.

We observed massive H3K27ac and less pronounced H3K4me3 chromatin changes during ST differentiation, confirming the results of earlier *in vitro* and *in vivo* trophoblast studies (Ounadjela et al., 2024; Wang et al., 2024). Inhibition of the SWI/SNF activity during the ST differentiation severely affected H3K27ac levels and, to a lesser extent, H3K4me3. These findings are in agreement with a number of studies showing that genetic or chemical ablation of SWI/SNF activity results in widespread loss of H3K27ac, predominantly at enhancers compared to promoters (Alver et al., 2017; Martin et al., 2023; Ren et al., 2024). Interestingly, a recent report showed that SWI/SNF preferentially regulates enhancers of signalling, developmental and cell-identity genes as compared to housekeeping genes, which is in line with our results (Basurto-Cayuela et al., 2024). It has been well established that SWI/SNF cooperates with histone acetyltransferases at distal regulatory regions/enhancers, where it safeguards local chromatin accessibility and ensures cell-specific transcriptional outputs (Alver et al., 2017; Huang et al., 2003; Ren et al., 2024). The importance of SWI/SNF and histone acetyltransferase activities was also confirmed in the trophoblast context. Similar to our results demonstrating that SWI/SNF is essential for ST differentiation, inactivation of the histone acetyltransferase P300 (but not CREBBP) also abrogates ST differentiation (van Voorden et al., 2023).

Our observations indicate that SWI/SNF inhibition resulted in the failure of hTSCs to exit the cell cycle as they differentiated into ST. Similar findings were reported in other differentiation models, as well as in cancer. For example, in muscle progenitors, SWI/SNF acts in concert with the TF MyoD, which antagonizes Polycomb-mediated transcriptional repression and suppresses Cyclin E transcription to arrest cell division (Ruijtenberg and Van Den Heuvel, 2015). As in ST, cell cycle exit is crucial for rapid and terminal muscle differentiation. In another model, the *Caenorhabditis elegans* anchor cell invasion, the SWI/SNF has been implicated in the regulation of the G0 cell cycle arrest (Smith et al., 2022). However, the mitotic role of the SWI/SNF complex is not only limited to cell cycle exit control but also to a wider range of crucial processes during mitosis. These include decatenation of newly replicated sister chromatids (Dykhuizen et al., 2013), mitotic bookmarking to safeguard cell identity during cell division (Zhu et al., 2023) as well as the resolution of transcription-replication conflicts (Bayona-Feliu et al., 2021), protecting genome stability. Similarly, our results suggest that the SWI/SNF complex plays a role not only in G1 exit during differentiation, but also during the G2/M chromatid stage. This dual functionality may explain why SWI/SNF inhibition promotes progression through G1/S, yet ultimately impairs overall cell proliferation, underscoring the need for careful dissection of multifunctional chromatin-remodelling complexes.

Developmental cell fate transitions are often driven by master TFs in cooperation with chromatin remodelling and modifying complexes. Here, we have demonstrated that while GCM1, CEBPB and TBX3 are each necessary for ST differentiation, only GCM1 is sufficient to induce it. Consistently, we found that GCM1 interacts with the SWI/SNF complex and shares a set of target chromatin regions, indicating that the CT to ST transition is coordinately regulated by GCM1 and the SWI/SNF. GCM1 is expressed almost exclusively in the placenta and has been established as one of the earliest master regulators of ST in both murine and human contexts. Mouse *Gcm1* null mutants die by E10.5 due to severe placental defects caused by the lack of a labyrinth compartment and the failure to form the ST (Anson-Cartwright et al., 2000). In the human context, the depletion of *GCM1* abrogates not only ST but also EVT differentiation (Jeyarajah et al., 2022). In both species, GCM1 is considered an upstream regulator of a subset of ST-specific genes (Anson-Cartwright et al., 2000; Jeyarajah et al., 2022; Schubert et al., 2008), in line with our findings that forced expression of GCM1 in basal media induces ST markers. These observations suggest that GCM1 cooperates with SWI/SNF to serve as a pioneer TF. Pioneer TFs facilitate chromatin accessibility at otherwise inaccessible regions of the genome by binding their target motifs and evicting nucleosomes through either steric mechanisms or the activity of chromatin remodelling enzymes they cooperate with (Balsalobre and Drouin, 2022). Such a mechanism operates in mESCs, where SWI/SNF continuously restores local accessibility of chromatin and enables binding of the pioneering TF Oct4 at the distal regulatory regions. This, in turn, facilitates the binding of other TFs, including Nanog and Sox2, and sustains the gene regulatory network driving ESC pluripotency and self-renewal (Iurlaro et al., 2021; King and Klose, 2017). Similarly, Eomes cooperates with the SWI/SNF complex to rewire chromatin and establish enhancer accessibility as the prerequisite for mesendoderm-specific gene expression during mESCs differentiation (Schröder et al., 2025). Future work will illuminate whether pioneer TFs stabilize recruitment or only enable SWI/SNF action on lineage-specific enhancers in the trophoblast context, in line with recently proposed mechanistic models (Ahmad et al., 2024; Zaret, 2024).

Taken together, our findings highlight a dual role for the SWI/SNF complex in the trophoblast: first, in facilitating G1/G0 cell cycle exit – a prerequisite for ST differentiation; and, second, in driving the transcriptional program necessary for this process. The underlying mechanisms involve epigenetic regulation of a large number of loci and genes during SR to ST differentiation without massive changes in SWI/SNF chromatin occupancy. Instead, SWI/SNF cooperates with GCM1 to initiate the GCM1-dependent ST transcriptional program, while subsequent interactions with additional TFs, such as TBX3, further reinforce and sustain ST differentiation.

### Study limitations

This study demonstrates that the SWI/SNF remodelling complex is indispensable for ST differentiation, a vital event during early human placental development. While we followed expression and chromatin changes during the early stages of ST differentiation and functionally tested several transcription factors, we did not specifically investigate the molecular mechanism underlying the ST differentiation. Future studies will be required to dissect the order of events and test whether GCM1 or another transcription factor recruits the SWI/SNF complex to ST-specific enhancers and drives ST differentiation.

## MATERIALS AND METHODS

### Human trophoblast stem cell culture

Human trophoblast stem cell (hTSC) lines CT30 and CT27 were gifted by Dr Hiroaki Okae (Tohoku University, Japan), authenticated and regularly tested for contamination. The cells were maintained, passaged and differentiated as reported by Okae et al. (2018). Minor modifications were applied; in short, fibronectin (Merck, FC010) was reconstituted in PBS to a final concentration 10 µg/ml, and plates/dishes were coated at 37°C for at least 60 min. The cells were cultured in hTSC base media (DMEM:F12; ThermoFisher, 11320074), 1×ST-X supplement (ThermoFisher, 51500056), 1.5 μg/ml L-ascorbic acid (Sigma-Aldrich, A4403) and 0.1 mM 2-mercaptoethanol (ThermoFisher, 31350010) supplemented with 0.2% FBS (PAA laboratories, A15-108), 100 ng/ml EGF (R&D/Tocris, 236-EG), 3 µM CHIR99021 (R&D/Tocris, 4423), 1 µM A83-01 (R&D/Tocris, 2939) and 8 µM Y27632 (R&D/Tocris, 1254) to achieve hTSC full (SR) media. The cells were cultured at 37°C, and in 5% CO_2_, the media was changed every 2 days. The cells were passaged at ∼80% confluency at a 1:4 to 1:6 ratio, depending on the experimental setup. TrypLE (ThermoFisher, 12605028) was used for cell dissociation at 37°C and in 5% CO_2_ for 11 min. ST differentiation was induced with hTSC base media supplemented with 10 µM Y27632 (R&D/Tocris, 1254), 4% KnockOut Serum Replacement (ThermoFisher, 10828010) and 2 µM forskolin (BioGems, 6652995) (ST media) for 72 h. 3D differentiation of the ST was induced in suspension in hTSC base media supplemented with 10 µM Y27632 (R&D/Tocris, 1254), 4% KnockOut Serum Replacement (ThermoFisher, 10828010), 2 µM forskolin (BioGems, 6652995) and 50 ng/ml EGF (R&D/Tocris, 236-EG) for 72 h. Trophoblast organoids were cultured as described previously (Haider et al., 2024, 2018).

### Primary cultures

Isolation of CT for villous CT (vCT) 2D culture was carried out in collaboration with Dr Sandra Haider and Prof. Dr Martin Knöfler (Department of Obstetrics and Gynecology, Reproductive Biology Unit, Medical University of Vienna, Austria). Utilization of tissues and all experimental procedures were approved by the Medical University of Vienna ethics boards (Nr. 084/2009) and required written informed consent. Samples of first-trimester placenta tissues (6th to 12th week of gestation) were obtained from elective pregnancy terminations. The procedures for vCT 2D culture have been described in detail by Haider et al. (2024). In short, a first-trimester CT was isolated by Percoll gradient centrifugation and cultured as described in the section ‘Human trophoblast stem cell culture’. vCT was cultured on 20 µg/ml fibronectin (Merck, FC010) in Advanced DMEM/F12 (ThermoFisher, 12634028), supplemented with 10% FBS (PAA laboratories, A15-108). The cells spontaneously differentiate into ST within 72 h.

### FUCCI flow cytometry

A fluorescence ubiquitin cell cycle indicator (FUCCI) hTSC cell line was generated via lentiviral transduction using the plasmid pBOB-EF1-FastFUCCI-Puro (Addgene plasmid #86849). Lentiviral particles were produced by standard packaging in HEK293T cells, and the viral supernatant was collected 48 h post-transfection. For transduction, hTSCs were incubated with the viral supernatant containing the FUCCI construct for 16-18 h, followed by puromycin selection. The cells were cultured in SR or ST media, treated with 10 μM BBinh for 72 h and harvested using TrypLE as described above. Next, the cells were fixed with 4% paraformaldehyde/PBS at 4°C for 20 min. Immediately before the flow cytometry analysis, the cells were strained using a 100 μM cell strainer. CytoFLEX2 (Beckmann Coulter) was used for analysis.

### Paraffin wax embedding and tissue sectioning

Paraffin wax embedding of tissues was carried as described previously (Haider et al., 2024). In short, the tissues were fixed in 4% paraformaldehyde/PBS at 4°C for 4-24 h. Next, the tissues were washed with 70% ethanol, a 70% ethanol and Alcian Blue (Sigma-Aldrich, 33864-99-2) mixture, 96% ethanol, 100% ethanol and xylene (Sigma-Aldrich, 106-42-3). The tissues were impregnated with Histowax at 65°C in metallic moulds and transferred to a cold plate for solidification. The sectioning was carried out using a microtome (ThermoFisher, HM355S). The sections were depleted of paraffin wax and rehydrated, and antigen retrieval was carried out in citrate buffer (pH 6) (Sigma-Aldrich, 6132-04-3) for 20 min in a steam boiler (approximately 95°C). Next, a general immunofluorescence protocol was used for staining and imaging.

### Genetic modifications and inhibitors

Lentiviral knockdowns (KDs) were performed as described previously (Hornbachner et al., 2021). Briefly, short hairpin RNAs (shRNA) and no template control were cloned into pLKO.1-neo construct (Addgene plasmid #13425). The newly cloned constructs were transfected using Lipofectamine 3000 (ThermoFisher, L3000008) into HEK293T cells together with psPAX2 (gag/pol-pro for virus packaging) and pMD2.G (VSV-G for G envelope protein). The supernatant containing the virally produced KDs construct was collected after 48 h and used for hTSC transduction for 16-18 h. The selection was carried out using 350 µg/ml neomycin G418 (Sigma Aldrich, A1720). shRNA sequences are listed in Table S4. Inducible overexpression (iOX) was generated by cloning the coding sequence of gene of interest into PiggyBac-Tre-Dest-rTA-HSV-neo (gifted by Dr Joerg Betschinger, FMI Basel, Switzerland). The newly cloned constructs were transfected into hTSCs with a NEON transfection system kit (ThermoFisher, 10704289) and selected with 350 µg/ml neomycin G418 (Sigma-Aldrich, A1720). The overexpression was induced with 1 µg/ml doxycycline (Sigma-Aldrich, D3447). For the inhibition of the SWI/SNF complex, a dual BRM/BRG1 ATPase inhibitor BRM014 (MedChemExpresss, HY-119374) was used as described previously (Papillon et al., 2018) at 10 µM concentration.

### Protein isolation and western blot

Whole-cell protein lysates were harvested using TG buffer [20 mM Tris-HCl (pH 7.5), 137 mM NaCl, 1 mM EGTA, 1% Triton X-100, 10% glycerol,1.5 mM MgCl_2_ and 1x complete EDTA-free Protease inhibitor cocktail (Sigma-Aldrich, 5056489001)]. Protein concentration was determined using a Bradford assay (Bio-Rad, 5000006). 10-20 µg of protein was used per gel. The lysates were boiled in 1× Laemmle buffer [0.25 M Tris (pH 6.8), 0.28 M SDS, 40% glycerol, Bromophenol Blue, 10% 2-mercapto-ethanol] for 5 min. The boiled lysates were loaded on SDS-PAGE gels and run at 14 mA per gel in an SDS running buffer (0.25 M Tris base, 1.925 M glycine, 0.035 M SDS, pH adjusted to 8.3). Transfer was carried out on ice in ice-cold transfer buffer (25 mM Tris, 192 mM glycine and 10% methanol) at 250 mA for 90 to 120 min onto Immobilon-PVDF Membrane (Merck Millipore, IPVH00010). The membranes were blocked and stained using the primary antibody in 5% milk-PBS and 0.1% Tween20 at 4°C overnight. On the next day, the membranes were stained with secondary anybody in 5% milk-PBS and 0.1% Tween20 at room temperature for 1-2 h and imaged using Clarity Western ECL Substrate and Fusion-FX (Vilber Lourmat). Detailed information on antibodies is provided in Table S4.

### Co-immunoprecipitation assay

The whole-cell lysates were harvested in Hunt buffer [20 mM Tris/HCl (pH 8.0), 100 mM NaCl, 1 mM EDTA, 0.5% NP-40) supplemented with 1× complete EDTA-free Protease inhibitor cocktail (Sigma-Aldrich, 5056489001). M2-Flag beads (Sigma-Aldrich, M8823-5ML) were pre-washed three times with Hunt buffer. 80 μl of M2-Flag beads were added to 1 mg of whole-cell lysates and incubated overnight at 4°C rotating. Next day, the beads were washed twice with TBS for 5 min and once for 15 min rotating at 4°C. The purified proteins were eluted from the beads in 1×SDS loading dye by heat incubation at 95°C for 5 min. The eluate was used for western blotting as described above.

### RNA isolation and RT-qPCR

RNA extraction was carried out using RNeasy mini/micro and DNAse I kit (all from QIAGEN) according to the manufacturer's instructions. RNA quantification was carried out by Nanodrop (ThermoFisher). 1-3 μg of RNA was used for cDNA synthesis using random hexameric primers (ThermoFisher, SO142) and RevertAid Reverse transcriptase protocol (ThermoFisher, EP0442). qPCR was performed with the GoTaq qPCR master mix (Promega, A6002). qPCR was performed in technical triplicates, and PBGD was used as a housekeeping gene for normalization. The results are shown as means of *n* biological replicates. GraphPad Prism (version 10.4.1) was used for analysis and visualization. Statistical significance was determined using a two-tailed unpaired *t*-test or ordinary one-way ANOVA (****P<0.0001, ***P<0.001, **P<0.01, *P<0.05; ns, not significant). Primer sequences are provided in Table S4.

### Immunofluorescence

The cells were fixed with 4% paraformaldehyde/PBS at 4°C for 20 min, permeabilized with PBS-0.1%TritonX at room temperature for 15 min, and blocked in 4% donkey serum-PBS-0.1%TritonX at room temperature for 30 min. The cells were stained with primary antibodies in 4% donkey serum-PBS-0.1%TritonX at 4°C overnight. On the next day, the cells were stained with secondary antibody in 4% donkey serum-PBS-0.1%Tween20 at room temperature for 1-2 h. DAPI (4,6-diamidino-2-phenylindole) counterstain or Vectashield Vibrance Antifade Mounting Medium with DAPI was used. 10 μM of BrdU (5-bromo-2′-deoxyuridine) thymidine analog (Abcam, ab142567) was added to the hTSC culture media 24 h prior to harvesting and further processing. Detailed information on antibodies is provided in Table S4. Zeiss Imager A2 microscope with Zen 2012 software was used for imaging.

### 3′ RNA-seq

RNA extraction was carried out using RNeasy mini/micro and DNAse I kit (all from QIAGEN) according to the manufacturer's instructions. RNA quantification was carried out by Nanodrop (ThermoFisher) or RNA broad-range QUBIT assay kit (ThermoFisher, Q10211). Libraries were prepared using QuantSeq 3′ mRNA-Seq Library Prep Kit FWD for Illumina - Dual Indexing, 96 UDI (Workflow B), Set B1 (Lexogen) following the manufacturer's instructions. Quality control, pooling and sequencing were carried out in collaboration with the Vienna BioCenter Core Facilities (VBCF) Next Generation Sequencing (NGS) department.

### ATAC-seq

Assay for transposase-accessible chromatin (ATAC)-seq was performed according to Grandi et al. (2022). Prior to the cell harvest, the cells were treated with DNase I (Worthington, LS002006; final concentration 200 Kunitz units/ml) for 30 min at 37°C. Next, the cells were harvested using TrypLE, as described above, washed with PBS and counted, and 50,000 cells were aliquoted into DNA LoBinding tubes (Eppendorf, EP0030108051). The cell pellet was resuspended in ice-cold ATAC-seq Lysis Buffer [10 mM Tris (pH 7.5), 10 mM NaCl, 3 mM MgCl_2_, 0.1% Nonidet P40, 0.1% Tween20, 0.01% digitonin] and incubated for 3 min on ice. The lysis was stopped by dilution with ice-cold ATAC-seq wash buffer [10 mM Tris (pH 7.5), 10 mM NaCl, 3 mM MgCl_2_ and 0.1% Tween20]. Nuclei were then pelleted and resuspended in a transposition mix [1×TD buffer and 2.5 µl TDE1 TD enzyme (both from Illumina, 20034198), 16.5 µl PBS, 0.01% digitonin, 0.1% Tween20]. The transposition reaction was incubated for 30 min at 37°C with shaking at 1000 rpm. The transposition reaction was terminated by the addition of five times the volume of DNA Binding Buffer from the DNA Clean and Concentrator-5 kit (Zymo Research, D4013), and cleaned up with DNA Clean and Concentrator-5 kit by following the manufacturer's instruction. The library amplification and barcoding were carried out using the NEBNext Ultra II DNA library preparation master mix (New England Biolabs, E7645) and purified with the DNA Clean and Concentrator-5 kit. The DNA content was quantified by QUBIT dsDNA HS assay kit (ThermoFisher, Q33230). Quality control, pooling and sequencing were carried out in collaboration with the Vienna BioCenter Core Facilities (VBCF) Next Generation Sequencing (NGS) department.

### ChIP-seq

Chromatin immunoprecipitation was performed as described previously (Lackner et al., 2023). The cells were fixed with 1% formaldehyde (ThermoFisher, 28908) for 7 min at room temperature. The reaction was quenched by 0.125 M glycine. The cells were harvested in PBS containing 1× complete EDTA-free Protease inhibitor cocktail (Sigma-Aldrich, 5056489001), centrifuged and resuspended in an ice-cold immunoprecipitation (IP) buffer. IP buffer was freshly made by a 2:1 mixture of SDS buffer [100 mM NaCl, 50 mM Tris (pH 8), 5 mM EDTA (pH 8), 0.5% SDS] and TritonX buffer [100 mM NaCl, 100 mM Tris (pH 8.6), 5 mM EDTA (pH 8) and 5% TritonX] and 1× complete EDTA-free Protease inhibitor cocktail. Sonication was performed using Bioruptor Pico (Diagenode) at 4°C for 23 cycles, 15 s ON/30 s OFF. 30 µg (for transcription factors) and 10 µg (for histone modifications) of chromatin (DNA equivalent) were immunoprecipitated at 4°C overnight with primary antibodies. Detailed information on antibodies is provided in Table S4. Protein G Dynabeads (Invitrogen, 10004D) were blocked in an immunoprecipitation buffer supplemented with 1 mg/ml BSA (New England Biolabs, B9000S) and yeast tRNA for 60 min at 4°C. Next, the beads were washed and added to the immunoprecipitation reaction for 3 h at 4°C. The samples were washed with low salt buffer [50 mM HEPES (pH 7.5), 140 mM NaCl, 1%TritonX, 1×complete EDTA-free Protease inhibitor cocktail], high salt buffer [50 mM HEPES (pH 7.5), 500 mM NaCl, 1%TritonX, 1×complete EDTA-free Protease inhibitor cocktail] and TE buffer. Each sample was eluted in 200 µl of ChIP elution buffer (100 mM NaHCO_3_ and 1%SDS) and de-crosslinked using 0.2 M NaCl at 65°C, with shaking at 600 rpm overnight. On the next day, the samples were treated with RNAse A (60 min at 37°C, 600 rpm) and Proteinase K (30 min at 65°C). The samples were purified with a MinElute PCR purification kit (Qiagen). The DNA content was quantified using a QUBIT dsDNA HS assay kit (ThermoFisher, Q33230). 10 ng of DNA per sample was used for library preparation using NEBNext Ultra II DNA library preparation master mix (New England Biolabs, E7645). Quality control, pooling and sequencing were carried out in collaboration with the Vienna BioCenter Core Facilities (VBCF) Next Generation Sequencing (NGS) department.

### Data analysis

#### Statistical analysis

Statistics, most visualizations and quantitative analyses were carried out in Rstudio (2023.06.0 Build 421) with R (4.3.3, R Core Team, 2024) and specialized packages, as found in the respective paragraphs. Exceptions are stated where used.

#### RNA quantification (3′ RNAseq-QuantSeq)

Libraries generated with the QuantSeq FWD kit (Lexogen) were sequenced on an Illumina NovaSeq SP SR100 XP (single read 100 bp). Reads were trimmed with bbduk (version 38.86) NOREF. Quality control was performed using fastQC (0.11.9, https://qubeshub.org/resources/fastqc). Transcripts were mapped to the human reference genome hg38 (GENCODE v38; Frankish et al., 2021) using STAR (2.7.3c; Dobin et al., 2013). After indexing with samtools (1.10; Li et al., 2009) reads in genes were counted using htseq (version 0.11.2; Anders et al., 2015). Genes were filtered for normalized row sum count of 10 and normalized counts >0 in at least three samples. Differential expression analysis was conducted with DESEqn (1.40.2; Love et al., 2014) using the Wald test and Benjamini-Hochberg correction for multiple testing. MA plots were generated with ggplot2 (3.4.4). For correlation analysis between BB inhibitor-treated cells and KD cells, LFC values, as derived by DESeq2, were plotted. PC loadings were sorted by variance and plotted with ggplot2 (3.4.4; Wickham, 2016).

#### Cluster analysis of the 3′ RNAseq data

Variance-stabilization transformed expression data of the top 3000 most variable genes were scaled by row, and distances were measured by dist from the stats package (4.3.3). fviz_nbclust of the factoextra (1.0.7; Kassambara and Mundt, 2020) package was used to determine optimal cluster number by the elbow method. Agnes of the cluster (2.1.6; Mächler et al., 2012) package was used for hierarchical clustering by the ‘ward’ method. Median expression line plots and boxplots were generated with ggplot2 using previously computed row scaled variance stabilized counts (from DESeq2 output). Significance levels in boxplots were computed using ggpubr (0.6.0; Kassambara, 2016) stat_compare_means with a Kruskal–Wallis test.

#### ATAC-seq analysis

Libraries were sequenced on an Illumina NovaSeq S4 PE150 XP (paired-end 150 bp), aiming for a sequencing depth of 200 million reads/sample. Raw reads were trimmed to 50 bp with CUTADAPT (2.8) in the paired-end mode and aligned to the human reference genome hg38 (GENCODE v38) with STAR aligner (2.7.5c) with parameters as described by Bentsen et al. (2020). Alignments were further processed using samtools (1.10). Mitochondrial reads, reads with MAPQ less than 20 and duplicates, as marked by markdup, were discarded. Peaks were called with Genrich in the ATAC mode. Specific parameters can be found in the respective sections. For bigwig generation, peaks were called on all replicates with Genrich in the default settings, reads were counted by DiffBind and the data were normalized to DBA_LIBSIZE_PEAKREADS. The normalization factors were used in deeptools bamCoverage to generate scaled bigwig files. For downstream visualization, the scaled bigwig files of the replicates were combined using averageBigwig of deeptools.

#### Differential accessibility analysis

ATACseq alignments were filtered for nucleosome-free regions by the deeptools alignmentSieve with --minFragmentLength 38 and --maxFragmentLength 100, and replicates were merged before calling peaks with Genrich in ATAC mode with -p 0.05 -a 20. The resulting peaksets of nucleosome-free regions (SR, ST, ST_BBinh) were used to generate a merged peakset, including all detected peaks. For read counting with DiffBind, we used the unfiltered alignments to use the maximum depth of the data. Counts were normalized to reads in peaks, as described above. Contrasts were directly defined, and analysis was run using DESeq2 with the internal RLE normalization. Differentially accessible regions were extracted using dba.report and annotated using the annotatePeak function of ChIPseeker (1.36.0, see below; Wang et al., 2022; Yu et al., 2015). Annotated peaks were used for downstream visualizations and integration with other data.

#### Transcription factor footprinting

Replicate (unfiltered) ATACseq alignment files for each condition were merged, and peaks were called with Genrich in ATACmode with -p 0.05 and -a 20. A consensus peak set with all peaks found in at least one sample was created by DiffBind. JASPAR2022 (Castro-Mondragon et al., 2022) motif files were downloaded, and only motifs of TFs that reach a normalized row sum count of 10 with a count >0 in at least 3 samples in our 3′mRNAseq (see above) were used in TOBIAS (Bentsen et al., 2020) FormatMotifs to generate the motif collection for footprinting. The TOBIAS footprinting pipeline (Bentsen et al., 2020) was run on the merged alignment files with the consensus peak set as peak regions. FootprintScores were generated from each condition and BINDetect was run with a BRG1/BRM merged consensus peak set to find differential transcription factor binding within all SWI/SNF target regions. Volcano plots of the BINDetect output were generated with ggplot2.

#### Chromatin immunoprecipitation sequencing

Libraries were sequenced on an Illumina NovaSeq S4 PE150 XP (paired-end 150 bp), aiming for a sequencing depth of 20-40 million reads/sample. Raw reads were trimmed with CUTADAPT (2.8; Martin, 2011) in the paired-end mode and aligned to the human reference genome hg38 (GENCODE v38; Frankish et al., 2021) with bowtie 2 (2.3.5.1; Langmead and Salzberg, 2012). Alignments were further processed using samtools (1.10). Mitochondrial reads, reads with MAPQ less than 20 and duplicates, as marked by markdup, were discarded. Peaks of SWI/SNF subunits were called using MACS3 (3.0.0b1) with a *P*-value cutoff of 0.1 (Zhang et al., 2008). High-confidence peaks were generated by IDR filtering (1.2), using a cut-off of FDR<0.05 across all replicates via the ChIPpeakAnno package (3.34.1) (Zhu et al., 2010). Peaks for histone modifications were called using Genrich (0.6.2) with -q 0.05 -a 500 and all replicates were included (H3K4me3, *n*=2; H3K27ac, *n*=2; BRG1, *n*=2; BRM, *n*=2). Separate replicated inputs were used as controls for each ChIP-seq dataset. A subset of the ChIP-seq samples was processed with the IMB/IMBA Vienna Biocenter Bioinfo core ChIPSeq pipline (v0.2) (contact ii-bioinfo@imp.ac.at IMP IMBA bioinformatics core, fork of NGI-ChIPseq v 1.4; C. Wang, P. Ewels).

#### ChIPseq quantification and visualization

ChIPseq quantification was carried out using DiffBind (3.10.1; Ross-Innes et al., 2012). Reads were counted across consensus peaksets for each ChIPseq dataset and normalized to background reads across the genome by setting background=TRUE during the counting step. Normalization factors were extracted using the dba.normalize function with the bRetrieve option. For visualization, we generated coverage files with deeptools (3.5.4; Ramírez et al., 2016) and bamCoverage using the normalization factors calculated in DiffBind. Heatmaps were generated by computeMatrix, resolving missing values as zeros and skipping zeros on the respective peak sets followed by the plotHeatmap command. Regions were sorted in descending order using the maximum signal. ChIP-seq tracks were visualized using Galaxy (Galaxy Community, 2022) and UCSC genome browser (Kent et al., 2002) custom tracks.

#### Differential ChIP enrichment analysis

Differential ChIP enrichment analysis was calculated using DiffBind. As a reference, we used the merged ATACseq peakset (see above). Counts in peaks were normalized to reads in peaks (see above). Contrasts were defined directly and analysed using DESeq2 with native RLE normalization and a Wald test with Benjamini-Hochberg multiple testing correction. MA plots were generated with ggplot2 (3.4.4; Wickham, 2016). Differentially enriched regions were extracted using dba.report and annotated using the annotatePeak function of ChIPseeker (1.36.0, see below; Wang et al., 2022; Yu et al., 2015). Annotated peaks were used for downstream visualizations and integration with other data.

#### Integration of ATAC-seq, ChIP-seq and 3′ RNAseq data

Peak overlaps and Venn diagrams of the ChIP/ATAC overlaps were prepared using the ChIPpeakAnno package. ATAC/ChIP integrative heatmaps were prepared using deeptools (see above) and show ChIP signal in the differentially accessible regions, as determined by ATAC-seq. Feature distribution of report files (DiffBind, see above) of ATAC-seq and ChIP-seq data was determined, and peaks were annotated with ChIPseeker (1.36.0, see below; Wang et al., 2022; Yu et al., 2015) with TxDb.Hsapiens.UCSC.hg38.knownGene (3.17.0; Bioconductor Core Team, 2017) and org.Hs.eg.db (3.17.0; Carlson, 2017) as AnnoDb and the following priorities: Promoter, 5′UTR, 3′UTR, Exon, Intron, Downstream, Intergenic. For integration of ATAC-seq and ChIP-seq signals with the 3′ RNAseq data, genomics data were quantified in the respective peak regions by DiffBind, as described above. All regions associated with a gene were considered when assigning the count values to the groups of genes defined by the 3′ RNAseq data. Median line plots and boxplots were generated with ggplot2 using scaled (by row/region) Conc values, as derived by DiffBind. Significance levels were computed by the stats_compare_mean function of ggpubr using a Kruskal–Wallis test and indicated in the figure (****P<0.0001, ***P<0.001, **P<0.01, *P<0.05; ns, not significant). After the extraction of specific gene sets, functional enrichment analysis was conducted as described below.

#### Functional enrichment analysis

Over-representation analysis (ORA) for GO terms (Ashburner et al., 2000; Gene Ontology Consortium et al., 2023) and KEGG (Kanehisa et al., 2023; Kanehisa and Goto, 2000) related terms was conducted with clusterProfiler (4.8.3; Wu et al., 2021; Yu et al., 2012). Over-representation analysis for the CellMarker_augmented_2021 database was conducted with EnrichR (Chen et al., 2013; Kuleshov et al., 2016; Xie et al., 2021). The results were plotted with the internal plotting function or ggplot2. ClusterProfiler and ErichR perform a one-sided Fisher's exact test for ORA. *P*-values of enrichment analyses were adjusted for multiple testing by the Benjamini-Hochberg method, and only terms with an adjusted *P*-value<0.05 were considered.

## Supplementary Material

10.1242/develop.204770_sup1 Supplementary information

Table S1. QuantSeq datasets. The table presents datasets of differentially expressed genes and gene clusters analysed in this study.

Table S2. ATAC-seq datasets. The table summarizes the ATAC-seq analysis results for SR, ST, and ST_BBinh conditions following 24h of differentiation and BBinh treatment.

Table S3. ChIP-seq datasets. The table presents the results of ChIP-seq analysis for BRM, BRG1, H3K27ac, and H3K4me3 in SR, ST, and ST_BBinh, as well as integration of TBX3 and CEBPB ChIP-seq with misregulated gene expression in corresponding knockdown lines.

Table S4. List of primers, shRNAs, and antibodies used in this study.
