# Supplementary material for: Activity of the SWI/SNF complex is indispensable for syncytiotrophoblast formation
Source: Development. 2025 Oct 31;152(21):dev204770. doi: 10.1242/dev.204770 (PMC12633796; doi:10.1242/dev.204770)
Supplement: Supplementary information [file develop-152-204770-s1.pdf]

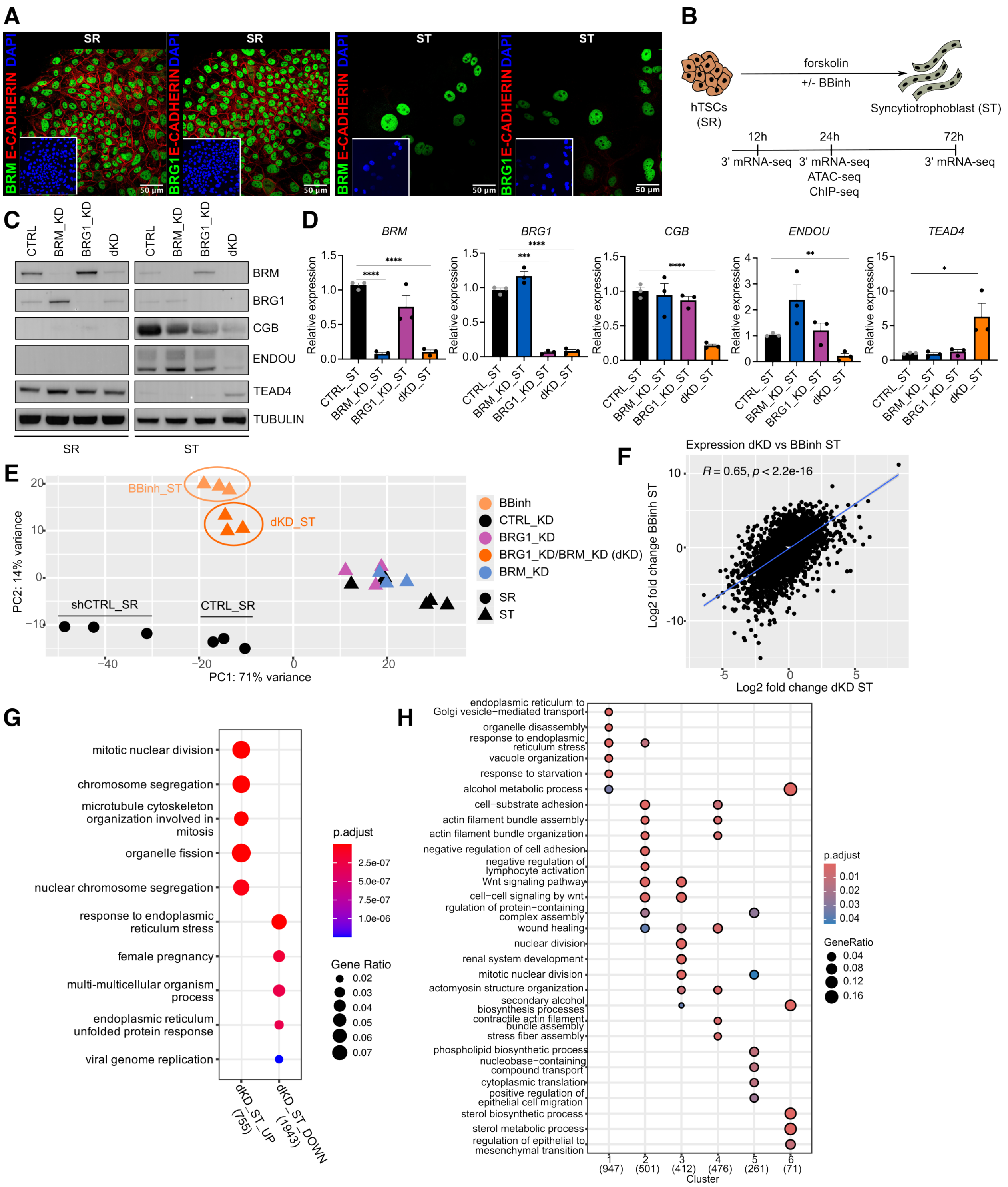

Development • Supplementary information

Fig. S1. BRM/BRG1 inhibition recapitulates BRM/BRG1 double KD.
A. Immunofluorescence staining of BRM or BRG1 (green), E-CADHERIN (red), and DAPI (blue) in hTSC and ST (72h). **B.** Experimental set-up: hTSC were cultured in SR or ST (forskolin) media with or without BBinh treatment for 12, 24, and 72h. The cells were harvested for 3'mRNA-seq for each of these time points. The cells from the 24h time point were additionally harvested for ATAC- seq and ChIP-seq. **C.** Western blot of single (BRM_KD, BRG1_KD) and double BRM/BRG1 KD (dKD) in hTSC in SR and ST conditions (72h). TUB (tubulin) serves as a loading control. **D.** RT- qPCR analysis of single KDs, dKD, and respective controls in hTSC in SR and ST (72h). Unpaired t-test (mean + SEM), n=3. **E.** PCA plot based on global expression (QuantSeq) in SR, ST, BBinh_ST, BRM_KD_ST, BRG1_KD_ST, and dKD_ST after 72h of differentiation/treatment (n=3). **F.** Correlation plot of BBinh_ST and dkD_ST expression from QuantSeq, Spearman correlation. **G.** Gene ontology terms for biological processes between dKD_ST and CTRL_ST (n=3). **H.** Gene ontology terms for biological processes for cluster groups from Figure 1J.

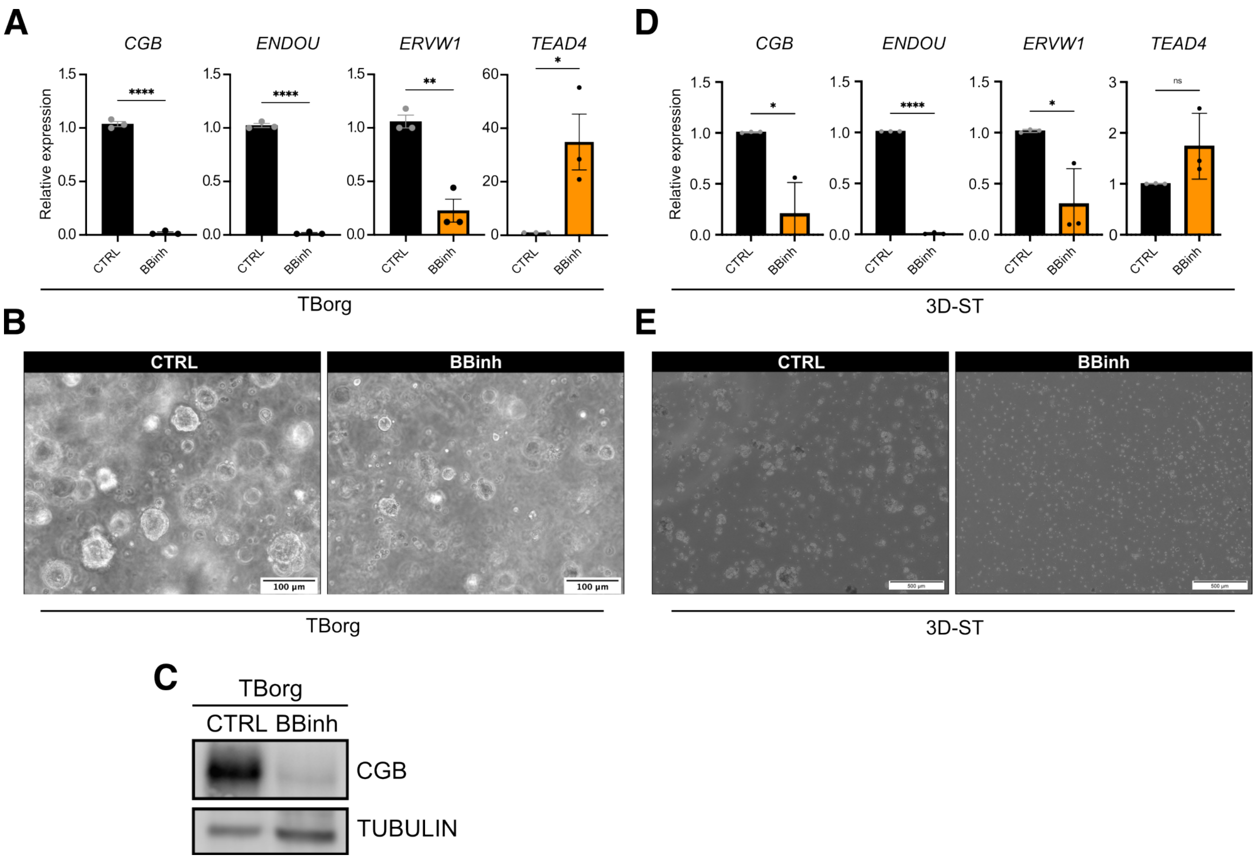

Fig. S2. BRM/BRG1 inhibition abrogates ST formation in TBorg and 3D- ST.
A. RT-qPCR analysis of trophoblast organoids (TBorg) derived from hTSCs after 3 days in BBinh or control (CTRL) conditions. Unpaired t-test (mean + SEM), n=3. **B.** Bright field images of TBorg after 3 days in BBinh or control conditions**.** Western blot in TBorg after 3 days in BBinh or control conditions showing CGB. TUBULIN serves as a loading control. **D.** RT-qPCR analysis of 72h 3D- ST differentiation in BBinh or control (CTRL) conditions. Unpaired t-test (mean + SEM), n=3. **E.** Bright field images of 72h 3D-ST differentiation in BBinh or control (CTRL) conditions.

Development • Supplementary information

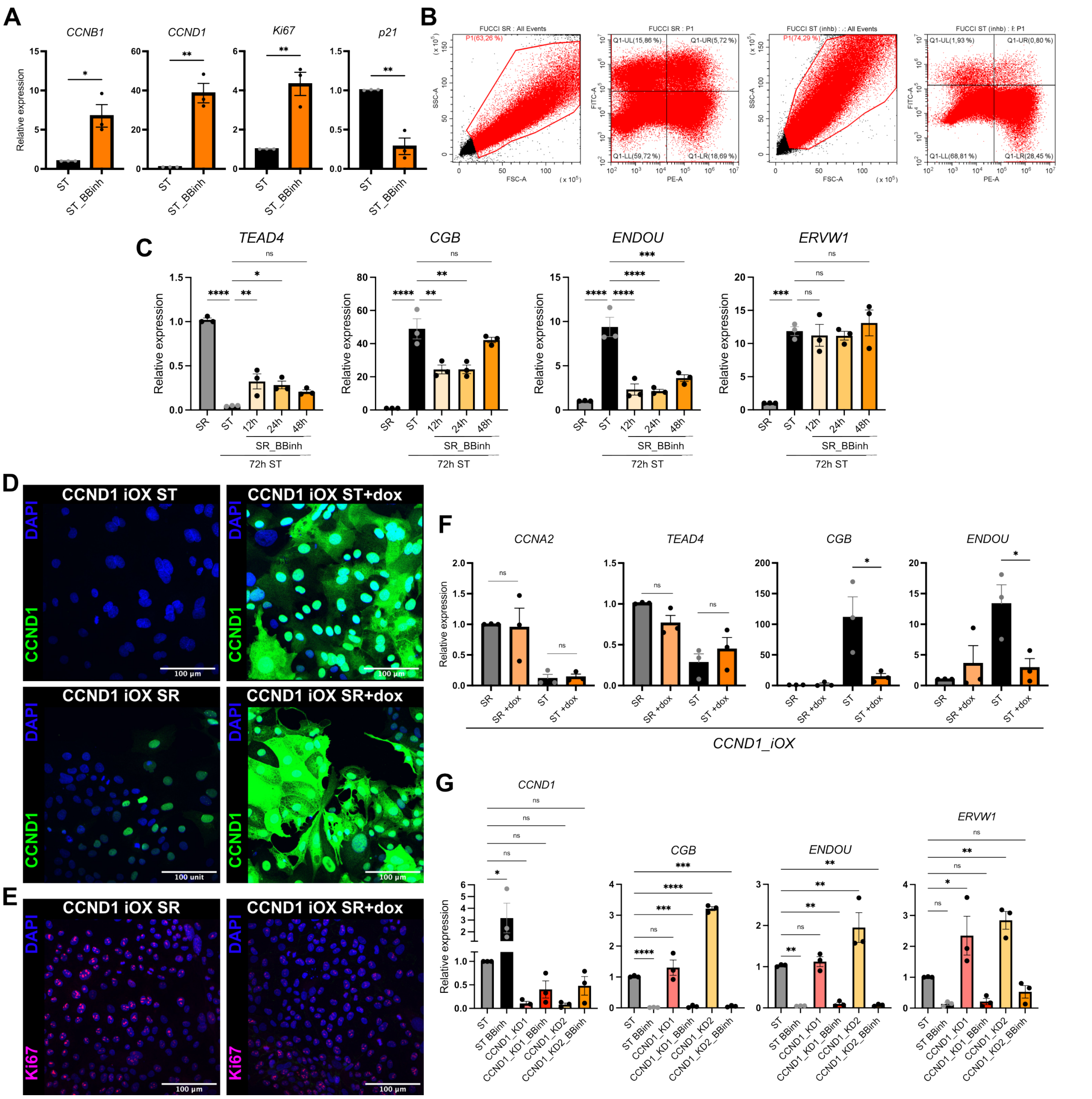

Fig. S3. Cell cycle misregulation and CCND1 contribute to ST differentiation failure. **A.** RT-qPCR analysis of cell cycle regulators in ST and BBinh_ST at 72h. Unpaired t-test (mean + SEM), n=3. **B.** Representative FACS plots and gating strategy of FUCCI cell line in SR and ST conditions at 72h. **C.** RT-qPCR analysis of 12, 24, and 48h BBinh_SR-primed hTSC, subsequently differentiated to ST without BBinh for 72h. Unpaired t-test (mean + SEM), n=3 **D.** Immunofluorescence of CCND1 (green), and DAPI (blue) analysis in CCND1 iOX hTSC in SR, SR +dox, ST, and ST+dox at 72h. **E.** Immunofluorescence of Ki67 (magenta) and DAPI (blue) analysis in CCND1 iOX hTSC in SR and SR+dox at 72h. **F.** RT-qPCR analysis of hTSC with CCND1 iOX construct in SR, ST, and ST+dox at 72h, n = 3 (unpaired t-test, mean +/- SEM). **G.** RT-qPCR analysis of cell cycle and differentiation markers in ST, CCND1_KD1, CCND1_KD2 in ST control condition and treated with BBinh at 72h, n = 3 (one way ANOVA, mean +/- SEM).

Development • Supplementary information

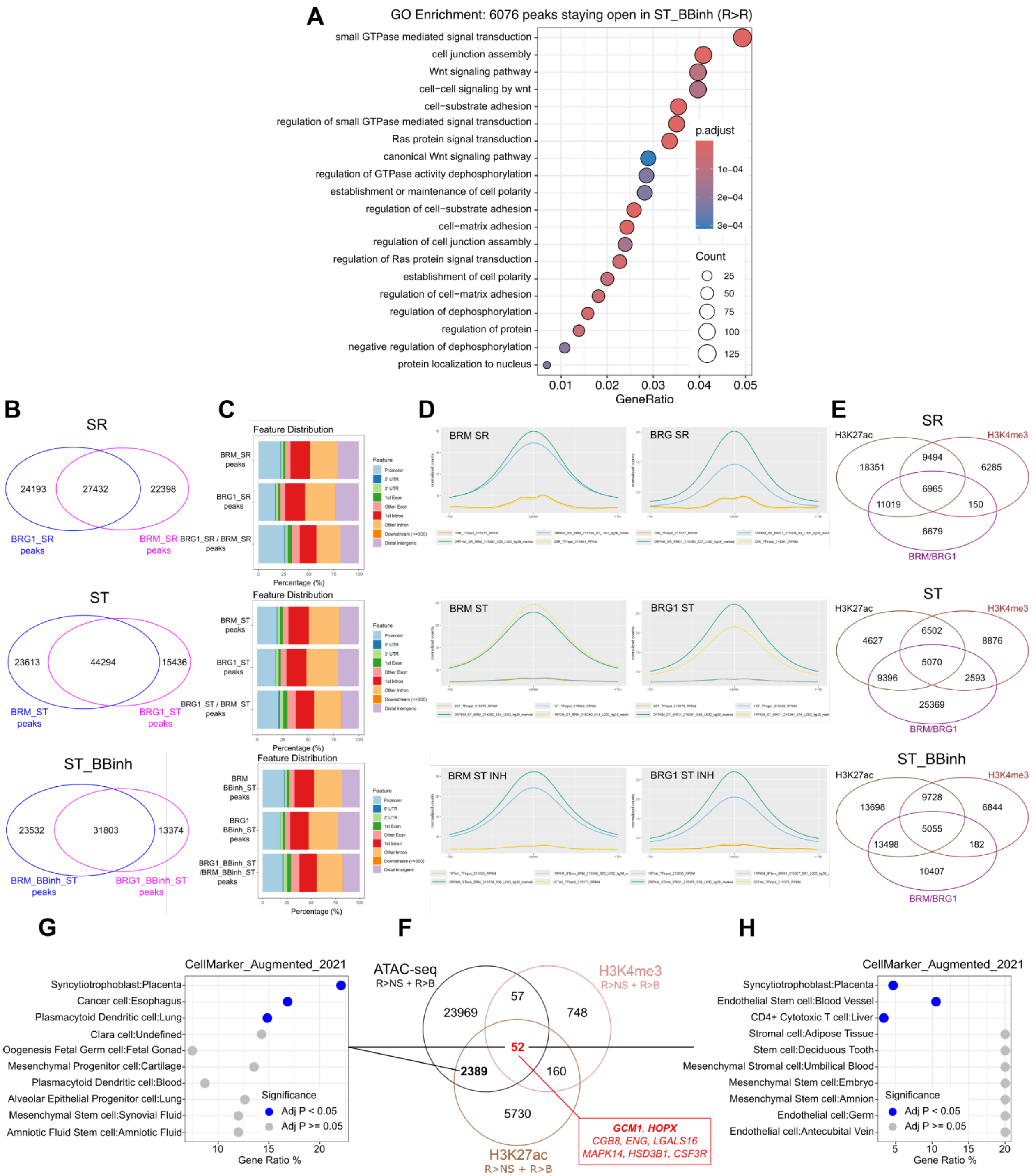

Development • Supplementary information

Fig. S4. Inhibition of BRG1/BRM function attenuates chromatin architecture during ST differentiation.

A. Gene ontology enrichment analysis on ATAC-seq regions that remain open in ST_BBinh (R>R) conditions. **B.** Venn diagrams showing the overlap of ChIP-seq peaks between BRG1- and BRM-bound regions in SR, ST, and ST_BBinh conditions. **C.** Feature distribution of regions occupied and co-occupied by BRG1 and BRM in SR, ST, and ST_BBinh conditions. **D.** RPKM normalized ChIP-seq profile plot for BRG1, BRM, and input (n=2) in SR, ST, and ST_BBinh conditions for the respective peak regions, generated by the vizzy R-package. **E.** Venn diagrams depicting the overlap of BRG1/BRM, H3K27ac, and H3K4me3 ChIP-seq peaks under SR, ST, and ST_BBinh conditions. (B-E) Analysis based on ChIP-seq peaks. **F.** Venn diagram showing the overlap between regions that failed to open in ST_BBinh (as defined by ATAC-seq, R>NS+R>B) and regions that failed to gain H3K27ac or H3K4me3 enrichment (R>NS+R>B) in ST_BBinh. Only promoter regions were selected for the analysis. **G.** EnrichR analysis of genes associated with overlapping regions between ATAC-seq and H3K27ac from F. **H.** EnrichR analysis on genes associated with overlapping ATAC-seq, H3K27ac, and H3K4me3 from F.

Development • Supplementary information

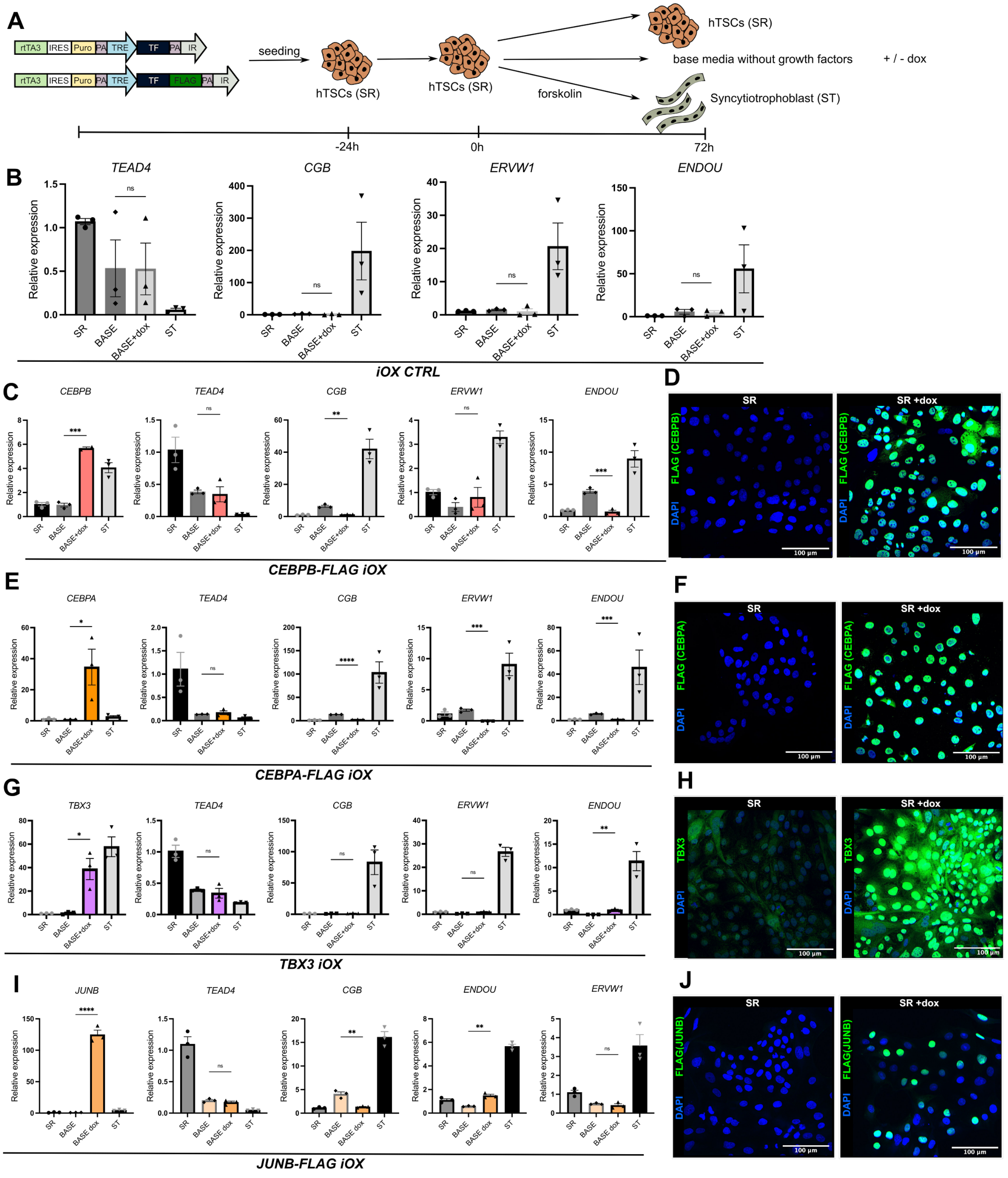

Development • Supplementary information

Fig. S5. CEBPB, CEBPA, TBX3, or JUNB do not drive the ST differentiation program. A. Design of the iOX experiments. **B.** RT-qPCR analysis of hTSC with control iOX construct in SR, base, base+dox, and ST at 72h, n = 3 (unpaired t-test, mean +/- SEM). **C.** RT-qPCR analysis of hTSC with CEBPB-FLAG iOX construct in SR, base, base+dox, and ST at 72h, n = 1 (unpaired t-test, mean +/- SEM performed on technical triplicates). **D.** Immunofluorescence staining of FLAG (green) and DAPI (blue) in hTSC with CEBPB-FLAG iOX construct in SR and SR+dox at 24h. **E.** RT-qPCR analysis of hTSC with CEBPA-FLAG iOX construct in SR, base, base+dox, and ST at 72h, n = 1 (unpaired t-test, mean +/- SEM performed on technical triplicates). **F.** Immunofluorescence staining of FLAG (green) and DAPI (blue) in hTSC with CEBPA-FLAG iOX construct in SR and SR+dox at 24h. **G.** RT-qPCR analysis of hTSC with TBX3 iOX construct in SR, base, base+dox, and ST at 72h, n = 1 (unpaired t-test, mean +/- SEM performed on technical triplicates). **H.** Immunofluorescence staining of TBX3 (green) and DAPI (blue) in hTSC with TBX3 iOX construct in SR and SR+dox at 24h.

I. RT-qPCR analysis of hTSC with JUNB-FLAG iOX construct in SR, base, base+dox, and ST at 72h, n = 1 (unpaired t-test, mean +/- SEM performed on technical triplicates). **J.** Immunofluorescence staining of FLAG (green) and DAPI (blue) in hTSC with JUNB-FLAG iOX construct in SR and SR+dox at 24h.

Development • Supplementary information

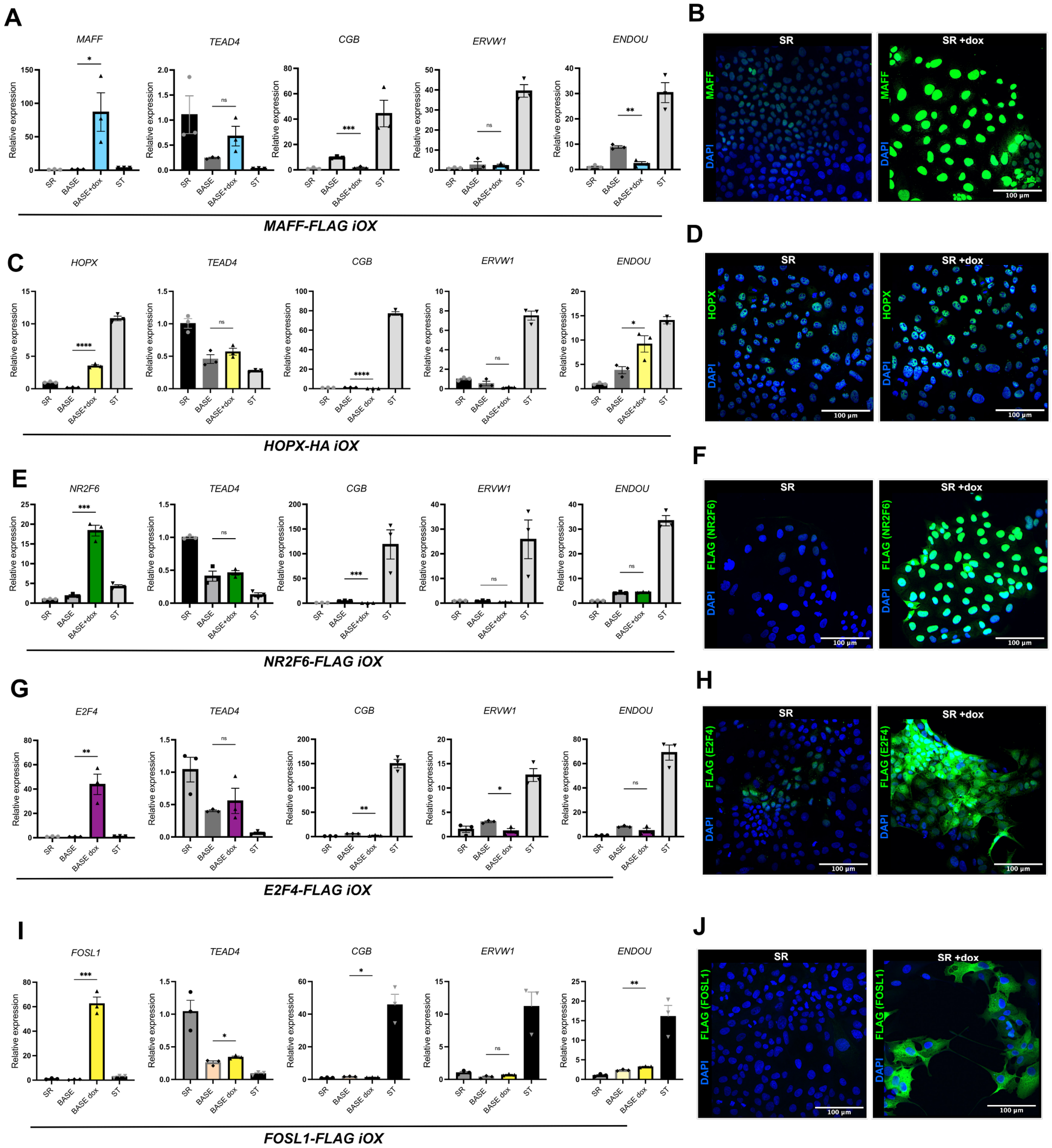
A
MAFF
TEAD4
CGB
ERVW1
ENDOU
MAFF-FLAG iOX
B
SR
SR +dox
C
HOPX
HOPX-HA iOX
D
E
NR2F6
NR2F6-FLAG iOX
F
G
E2F4
E2F4-FLAG iOX
H
I
FOSL1
FOSL1-FLAG iOX
J

Fig. S6. MAFF, HOPX, NR2F6, E2F4, or FOSL1 were not able to drive the ST differentiation program.

A. RT-qPCR analysis of hTSC with MAFF-FLAG iOX construct in SR, base, base+dox, and ST at 72h, n = 1 (unpaired t-test, mean +/- SEM performed on technical triplicates). **B.** Immunofluorescence staining of MAFF (green) and DAPI (blue) in hTSC with MAFF-FLAG iOX construct in SR and SR+dox at 24h. **C.** RT-qPCR analysis of hTSC with HOPX-HA iOX construct in SR, base, base+dox, and ST at 72h, n = 1 (unpaired t-test, mean +/- SEM performed on technical triplicates). **D.** Immunofluorescence staining of HOPX (green) and DAPI (blue) in hTSC with HOPX-HA iOX construct in SR and SR+dox at 24h. **E.** RT-qPCR analysis of hTSC with NR2F6-FLAG iOX construct in SR, base, base+dox, and ST at 72h, n = 1 (unpaired t-test, mean +/- SEM performed on technical triplicates). **F.** Immunofluorescence staining of FLAG (green) and DAPI (blue) in hTSC with NR2F6-FLAG iOX construct in SR and SR+dox at 24h. **G.** RT-qPCR analysis of hTSC with E2F4-FLAG iOX construct in SR, base, base+dox, and ST at 72h, n = 1 (unpaired t-test, mean +/- SEM performed on technical triplicates). **H.** Immunofluorescence staining of FLAG (green) and DAPI (blue) in hTSC with E2F4-FLAG iOX construct in SR and SR+dox at 24h. **I.** RT-qPCR analysis of hTSC with FOSL1-FLAG iOX construct in SR, base, base+dox, and ST at 72h, n = 1 (unpaired t-test, mean +/- SEM performed on technical triplicates). **J.** Immunofluorescence staining of FLAG (green) and DAPI (blue) in hTSC with FOSL1-FLAG iOX construct in SR and SR+dox at 24h.

Development • Supplementary information

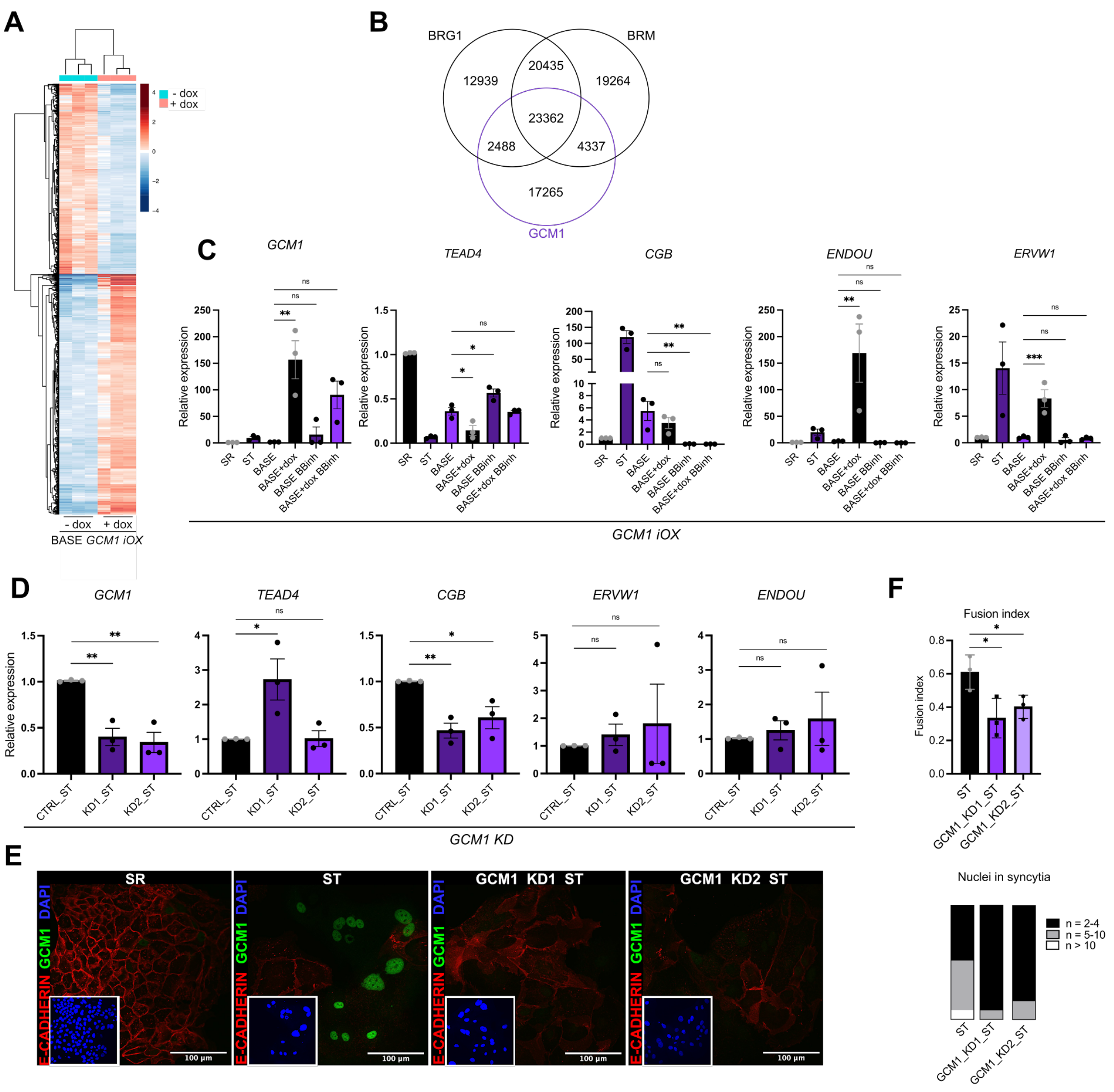

Fig. S7. GCM1 is a major driver of the ST transcriptional program.

A. Heatmap of differentially expressed genes between GCM1 iOX BASE+dox and BASE control (72h), (n=3, cutoff: |log2FC| > 1, p adj < 0.05). **B.** Venn diagram showing the overlap between regions bound by GCM1 and regions co-bound by BRG1 and BRM, as identified by ChIP-seq in (Shimizu *et al.*, 2023) and this study at 24h ST, respectively. **C.** RT-qPCR analysis of the GCM1- iOX cell line cultured under SR, ST, and BASE conditions, +/- dox and +/- BBinh. **D.** RT-qPCR analysis of GCM1_KD1, GCM1_KD2, and CTRL_KD in ST at 72h, n = 3 (unpaired t-test, mean +/- SEM). **E.** Immunofluorescence staining of GCM1 (green), E-CADHERIN (red), and DAPI (blue) in CTRL_SR, CTRL_ST, GCM1_KD1_ST, and GCM1_KD2_ST. **F.** Quantification of fusion index (fusion index = (number of nuclei in syncytia - number of syncytia) / total number of nuclei), syncytium was defined by 2 or more nuclei in GCM1_KD1_ST, GCM1_KD2_ST, and CTRL_ST. Unpaired t-test (mean + SEM), n=3, 3 areas, 2 independent observers.

Development • Supplementary information

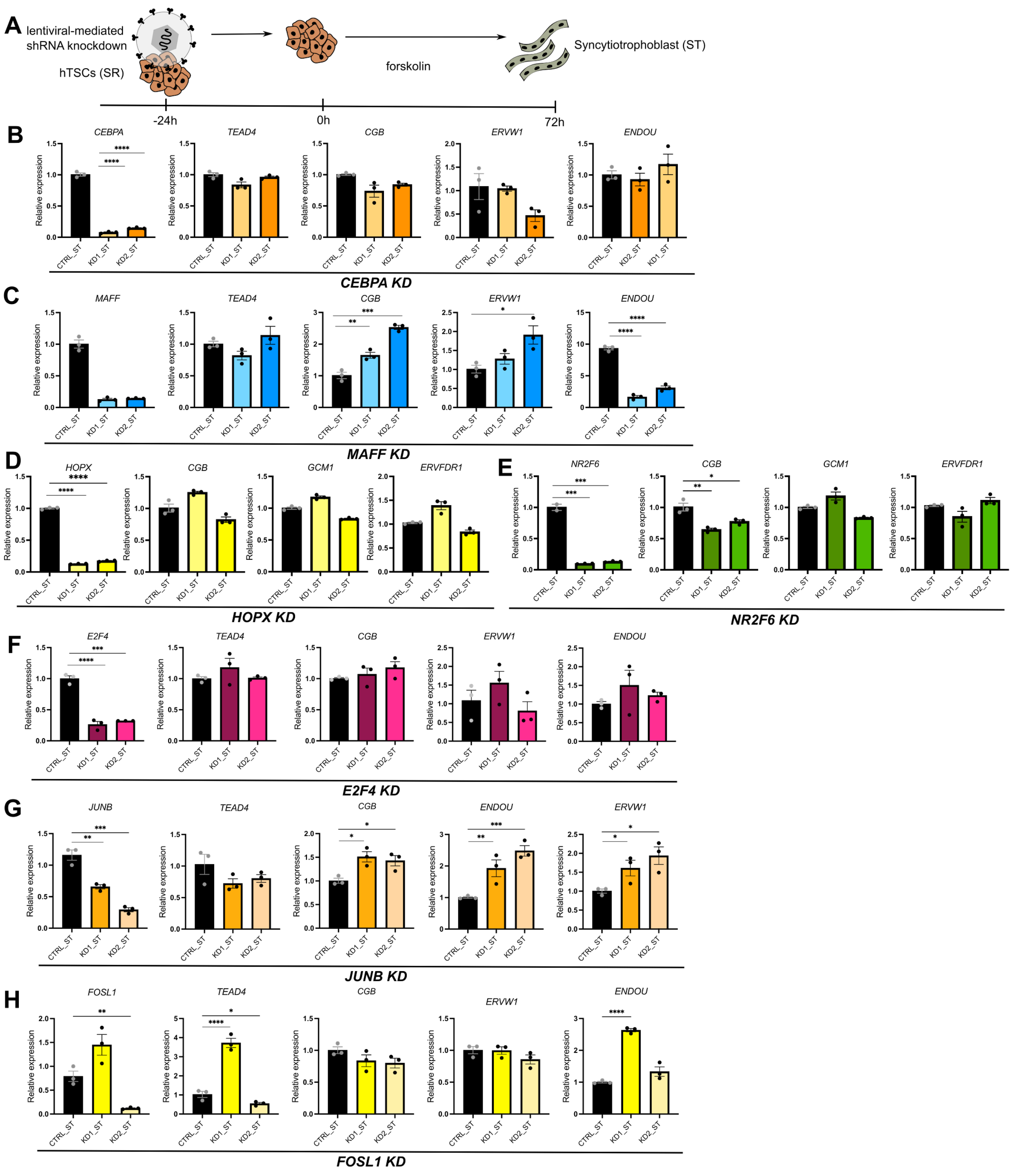

Development • Supplementary information

Fig. S8. CEBPA, MAFF, HOPX, NR2F6, E2F4, JUNB, or FOSL1 KDs do not cause ST differentiation defect.

A. Design of the KD experiments. **B.** RT-qPCR analysis of CEBPA KDs in ST at 72h, n = 1 (unpaired t-test, mean +/- SEM performed on technical triplicates). **C.** RT-qPCR analysis of MAFF KDs in ST at 72h, n = 1 (unpaired t-test, mean +/- SEM performed on technical triplicates). **D.** RT- qPCR analysis of HOPX KDs in ST at 72h, n = 1 (unpaired t-test, mean +/- SEM performed on technical triplicates). **E.** RT-qPCR analysis of NR2F6 KDs in ST at 72h, n = 1 (unpaired t-test, mean +/- SEM performed on technical triplicates). **F.** RT-qPCR analysis of E2F4 KDs in ST at 72h, n = 1 (unpaired t-test, mean +/- SEM performed on technical triplicates). **G.** RT-qPCR analysis of JUNB KDs in ST at 72h, n = 1 (unpaired t-test, mean +/- SEM performed on technical triplicates). **H.** RT-qPCR analysis of FOSL1 KDs in ST at 72h, n = 1 (unpaired t-test, mean +/- SEM performed on technical triplicates).

Development • Supplementary information

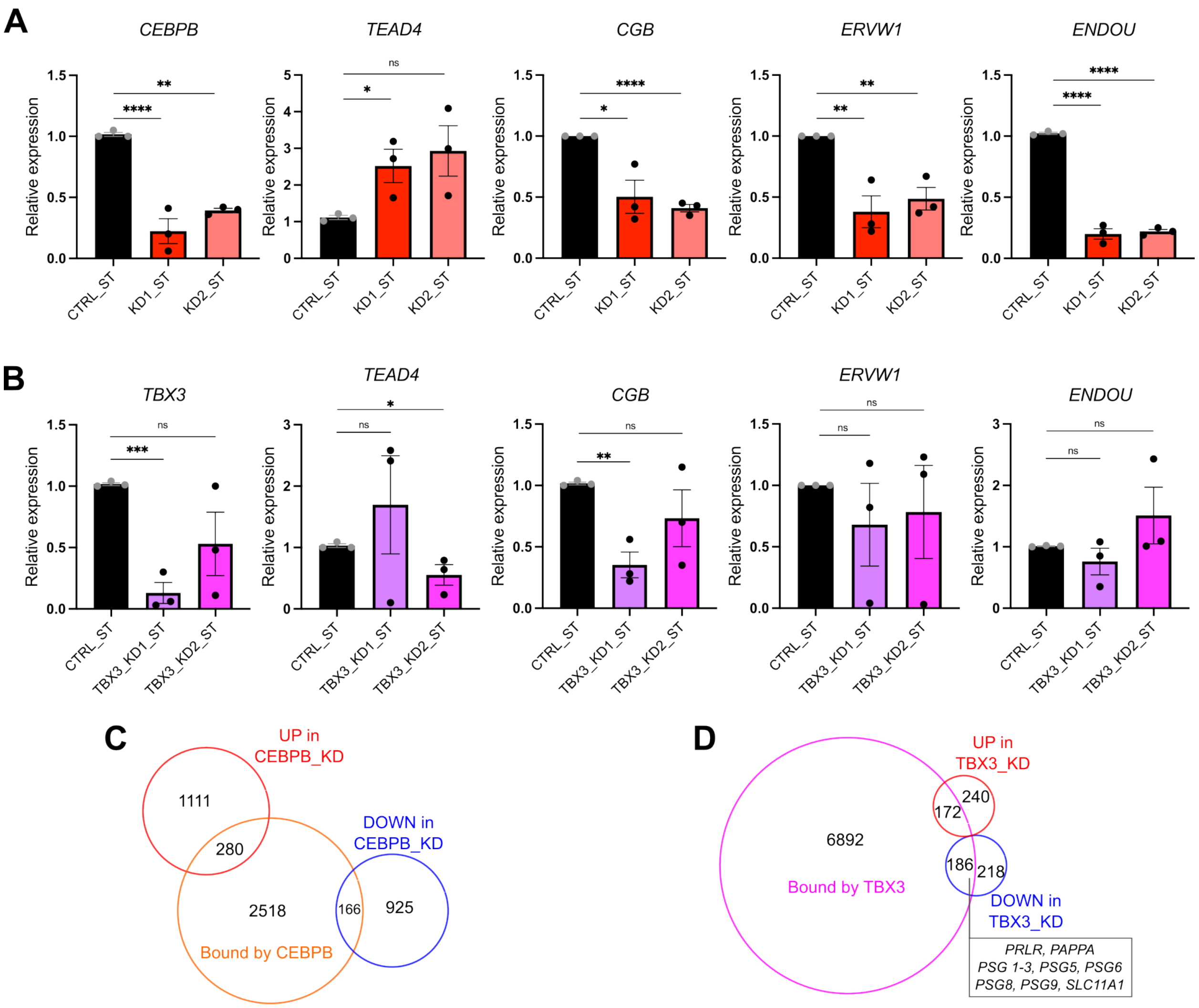

Fig. S9. CEBPB and TBX3 aid ST differentiation.

A. RT-qPCR expression analysis in two independent shRNA CEBPB KD lines (KD1 and KD2) and control KD line (CTRL) in ST at 72h, n = 3 (unpaired t-test, mean +/- SEM). **B.** RT-qPCR expression analysis in two independent shRNA TBX3 KD lines (KD1 and KD2) and control KD line (CTRL) in ST at 72h, n = 3 (unpaired t-test, mean +/- SEM). **C.** Venn diagram showing overlap of genes bound by CEBPB and those differentially expressed in the CEBPB KD line in ST. **D.** Venn diagram showing overlap of genes bound by TBX3 and those differentially expressed in the TBX3 KD line in ST. To annotate peaks to genes, the top 10000 peaks of TBX3 or CEBPB ChIP- seq were selected based on the top 25% fold enrichment and the top 75% q values.

Development • Supplementary information

Fig. 1B

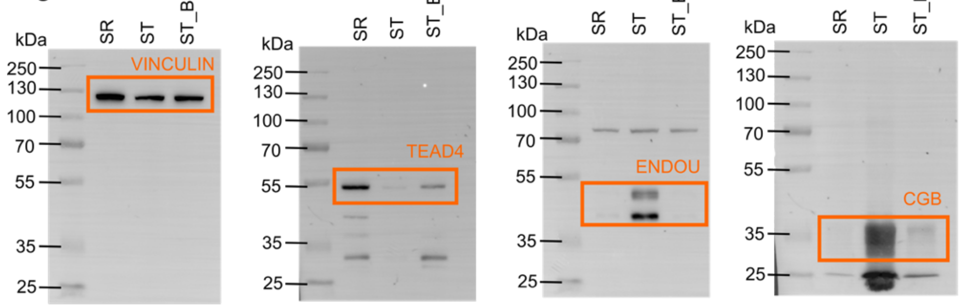

Fig. 1F

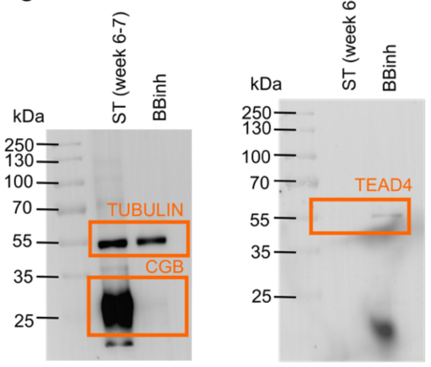

Fig. S1C

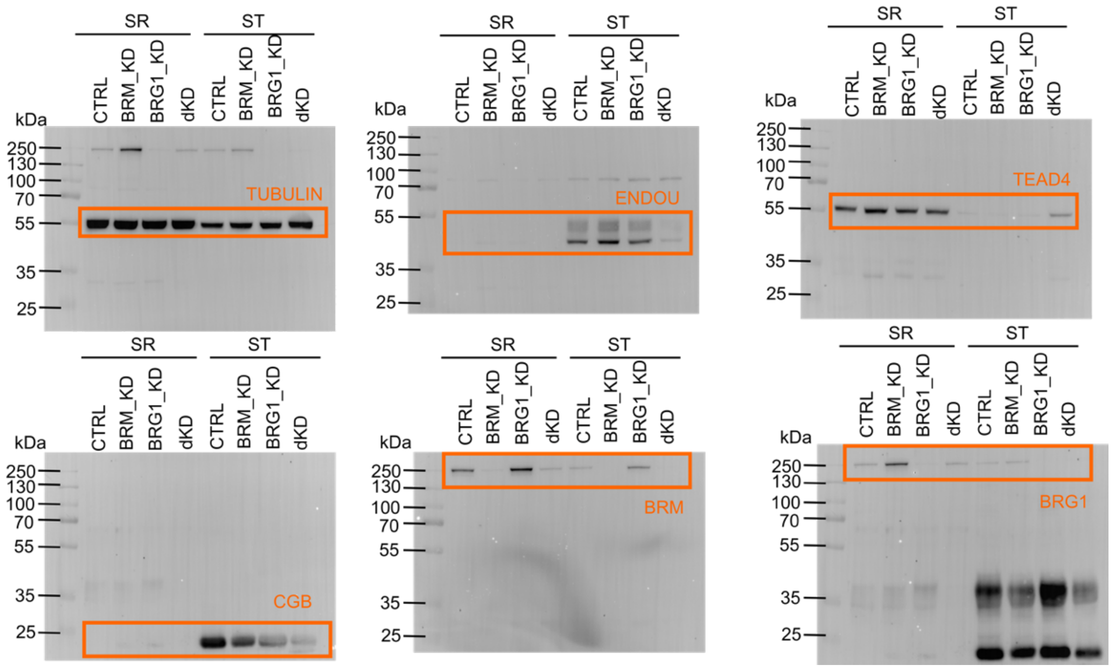

Development • Supplementary information

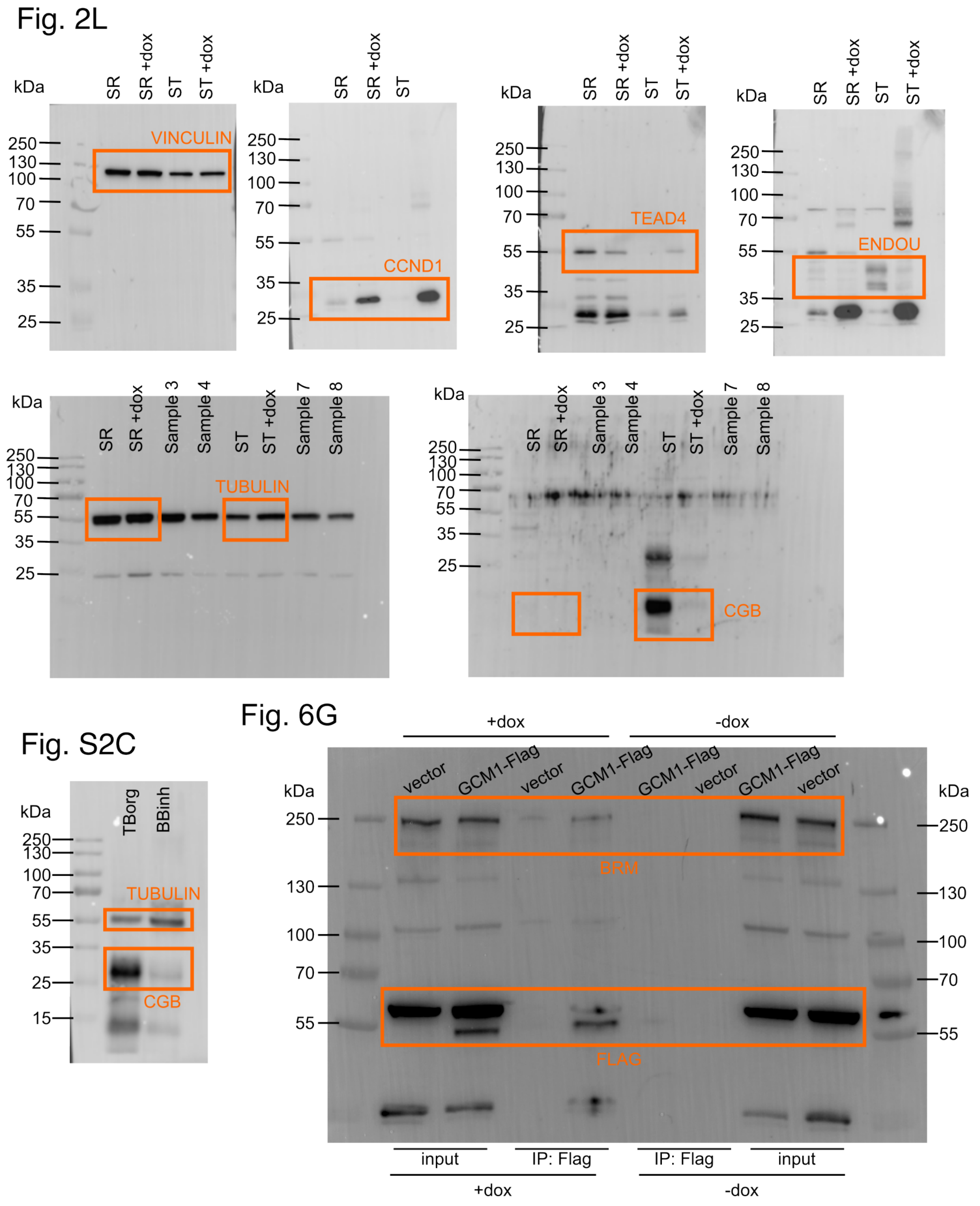

Fig. S10. Original Western blots.

The Figure presents original scans of the Western blot membranes corresponding to the indicated figures in this manuscript.

Development • Supplementary information

Table S1. QuantSeq datasets.

The table presents datasets of differentially expressed genes and gene clusters analysed in this study.

Available for download at
https://journals.biologists.com/dev/article-lookup/doi/10.1242/dev.204770#supplementary-data

Table S2. ATAC-seq datasets.

The table summarizes the ATAC-seq analysis results for SR, ST, and ST_BBinh conditions following 24h of differentiation and BBinh treatment.

Available for download at
https://journals.biologists.com/dev/article-lookup/doi/10.1242/dev.204770#supplementary-data

Table S3. ChIP-seq datasets.

The table presents the results of ChIP-seq analysis for BRM, BRG1, H3K27ac, and H3K4me3 in SR, ST, and ST_BBinh, as well as integration of TBX3 and CEBPB ChIP-seq with misregulated gene expression in corresponding knockdown lines.

Available for download at
https://journals.biologists.com/dev/article-lookup/doi/10.1242/dev.204770#supplementary-data

Table S4. List of primers, shRNAs, and antibodies used in this study.

Available for download at
https://journals.biologists.com/dev/article-lookup/doi/10.1242/dev.204770#supplementary-data

Development • Supplementary information
